# Real-world evidence of survival benefit of remdesivir: study of 419 propensity score-matched patients hospitalized over the alpha and delta waves of COVID-19 in New Orleans, LA

**DOI:** 10.3389/fmed.2024.1390164

**Published:** 2024-05-16

**Authors:** Nicolas Salvadori, Moshe Fridman, Mel Chiang, Linda Chen, ChenYu Wang, EunYoung Lee, Vivian Fonseca, Dahlene N. Fusco, Gonzague Jourdain, Arnaud C. Drouin

**Affiliations:** ^1^Department of Statistics, Faculty of Science, Chiang Mai University, Chiang Mai, Thailand; ^2^Faculty of Associated Medical Sciences, Chiang Mai University, Chiang Mai, Thailand; ^3^AMF Consulting, Los Angeles, CA, United States; ^4^Gilead Sciences, Inc., Foster City, CA, United States; ^5^Endocrinology Section, Department of Medicine, Tulane University School of Medicine, New Orleans, LA, United States; ^6^Department of Medicine, Tulane University School of Medicine, New Orleans, LA, United States; ^7^Department of Tropical Medicine, Tulane University School of Public Health and Tropical Medicine, New Orleans, LA, United States; ^8^University Medical Center, New Orleans, LA, United States

**Keywords:** COVID-19, remdesivir, real world experience, hospital mortality, propensity score-matched

## Abstract

**Background:**

The direct acting antiviral remdesivir (RDV) has shown promising results in randomized clinical trials. This study is a unique report of real clinical practice RDV administration for COVID-19 from alpha through delta variant circulation in New Orleans, Louisiana (NOLA). Patients in NOLA have among US worst pre-COVID health outcomes, and the region was an early epicenter for severe COVID.

**Methods:**

Data were directly extracted from electronic medical records through REACHnet. Of 9,106 adults with COVID, 1,928 were admitted to inpatient care within 7 days of diagnosis. The propensity score is based upon 22 selected covariates, related to both RDV assignment and outcome of interest. RDV and non-RDV patients were matched 1:1 with replacement, by location and calendar period of admission. Primary and secondary endpoints were, death from any cause and inpatient discharge, within 28 and 14 days after inpatient admission.

**Results:**

Of 448 patients treated with RDV, 419 (94%) were successfully matched to a non-RDV patient. 145 (35%) patients received RDV for < 5 days, 235 (56%) for 5 days, and 39 (9%) for > 5 days. 96% of those on RDV received it within 2 days of admission. RDV was more frequently prescribed in patients with pneumonia (standardized difference: 0.75), respiratory failure, hypoxemia, or dependence on supplemental oxygen (0.69), and obesity (0.35) within 5 days prior to RDV initiation or corresponding day in non-RDV patients (index day). RDV patients were numerically more likely to be on steroids within 5 days prior to index day (86 vs. 82%) and within 7 days after inpatient admission (96 vs. 87%). RDV was significantly associated with lower risk of death within 14 days after admission (hazard ratio [HR]: 0.37, 95% CI: 0.19 to 0.69, *p* = 0.002) but not within 28 days (HR: 0.62, 95% CI: 0.36 to 1.07, *p* = 0.08). Discharge within 14 days of admission was significantly more likely for RDV patients (*p* < 0.001) and numerically more likely within 28 days after admission (*p* = 0.06).

**Conclusion:**

Overall, our findings support recommendation of RDV administration for COVID-19 in a highly comorbid, highly impoverished population representative of both Black and White subjects in the US Gulf South.

## Introduction and background

SARS CoV-2 variants will likely circulate for years to come, underscoring an ongoing need for adequate guidelines and effective treatments independent of virus immunogenic shifts. To contain COVID-19 most effectively moving forward, a combined strategy of prevention (vaccines, intermittent masks, social distancing) and treatments [anti-inflammatory drugs, direct acting antivirals (DAAs), host directed antivirals (HDAVs), and passive immunotherapy in the form of antibodies] is needed (1, 2). Because of the extreme social vulnerability and health inequity among US populations, regionally focused Real-World Experience (RWExp) studies could inform local guidelines. A summary of clinical trials, observational studies, and the guidelines of the Infectious Diseases Society of America (IDSA) is provided to walk the reader out of the maze of conflicting results and guidelines released around use of RDV in COVID-19 patients and to provide rationale for the current study. Supplementary Table 1 provides the main characteristics and links to RDV randomized clinical trials (RCT) and retrospective studies, and Supplementary Table 2 provides IDSA as well as World Health Organization (WHO) and Food Drug Administration (FDA) guidelines for the use of RDV in COVID-19 mentioned in the next paragraphs, reported in the framework of COVID-19 waves, study RDV/non-RDV patient ratios and state’s vaccination of LA population over the study period (Figure 2). Pre-clinical phase studies: The direct acting antiviral RDV, or GS-5734, is a ribonucleotide analog inhibitor of viral RNA-dependent RNA polymerase. Rationale for the use of RDV for SARS CoV-2 came from *in vitro* and *in vivo* efficacy against Middle Eastern Respiratory Syndrome (MERS) CoV (3–7), *in vitro* activity against SARS CoV-2 (8) and *in vivo* efficacy of SARS CoV-2 in non-human primate studies (6, 9, 10).

**FIGURE 1:**
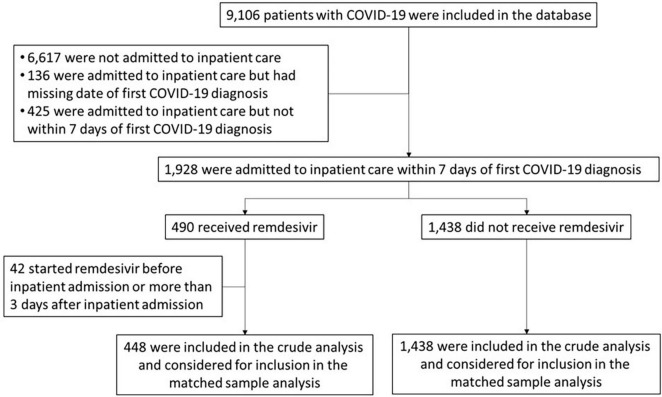
Study flow diagram.

**FIGURE 2:**
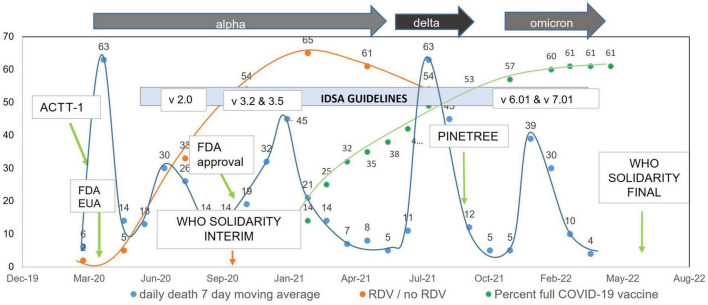
Remdesivir administration ratio and daily death 7-day moving average over time in the United States.

Early clinical trial, funded by the Chinese Academy of Medical Sciences Emergency Project of COVID-19: Early in the pandemic, from February to March of 2020, Wang et al. (11) performed a randomized, double-blind, placebo-controlled, multicenter trial at ten hospitals in Hubei, China to evaluate RDV in inpatients with COVID-19 (11). They compared 158 patients assigned to RDV to 79 patients assigned to placebo and found that RDV use was associated with numerically faster time to clinical improvement versus placebo among patients with symptom duration of 10 days or less, but this difference was not statistically significant. It is important to note that the study was underpowered due to enrollment challenges.

Early randomized clinical trial [ACTT-1] and study of compassionate use funded by Gilead Sciences, Inc.: In the early trial by Grein et al. (12) RDV under compassionate administration was beneficial in 53 patients with severe COVID-19. The preliminary results of the first randomized trial in the US, the ACTT-1 study, released in May 2020 in the COVID-19 pandemic, found RDV as a potentially useful therapeutic with favorable toxicity profile (13). In the ACTT-1 study, 541 patients were assigned to RDV and 521 to placebo, enrolled from February to April 2020 (Supplementary Table 1). The ACTT-1 population was 20% black, and 55% of participants had two or more coexisting conditions. ACTT-1 found that RDV was superior to placebo in shortening time to recovery in adults hospitalized with COVID-19 and with evidence of lower respiratory tract infection. The final result was communicated in October 2020 leading to RDV FDA Emergency Use Authorization (EUA) (14). Subsequent randomized clinical trials, SIMPLE and PINETREE, funded by Gilead Sciences, Inc.: The SIMPLE-Severe, (results released 1 June 2020) (15) trial compared 5 versus 10 days RDV in a randomized, open-label phase 3 trial of hospitalized patients with ambient air oxygen saturation ≤ 94% and radiologic evidence of pneumonia, finding no significant difference in 5 versus 10 day course for non-ventilated patients. SIMPLE-Moderate (16) was a randomized, open-label trial of 584 hospitalized patients with moderate COVID-19 pneumonia (pulmonary infiltrates and room-air oxygen saturation > 94%) enrolled from March 15 through 18 April 2020, at 105 hospitals in the United States, Europe, and Asia. SIMPLE-Moderate found that patients randomized to a 5-day, but not a 10-day, course of RDV had significant improvement in clinical status as measured on day 11. The later PINETREE study, released September 2021, showed that early treatment with three days of RDV prevented progression to severe COVID-19 in outpatients, including a population that was 7% black, 62% diabetic, and 55% obese (17). Two studies in April–May 2020 were combined to assess open label RDV: GS-5773 study (SIMPLE-Severe; oxygen ≤ 94, pulmonary infiltrates) and GS-5807 (a real-world control cohort). Because GS-5773 did not have a no treatment control arm, RDV efficacy was assessed by comparing results between 5,773 and 5,807 (no treatment), and this comparison found some survival benefit in RDV open label treatment group compared to the real-world control study group (18).

Randomized Clinical Trials, SOLIDARITY, sponsored by the WHO: The World Health Organization (WHO) led the SOLIDARITY clinical trial, presented as interim data in November 2020 (19) then final data in 2022 (20). SOLIDARITY is a multicenter international randomized adaptive clinical trial sponsored by the WHO to determine anti-COVID efficacy of multiple drugs selected over time. At various stages of the study, RDV has been compared to hydroxychloroquine, lopinavir, interferon β1a, or the tyrosine kinase inhibitor acalabrutinib, versus local standard of care, in patients hospitalized for SARS-CoV-2 infection. Outcome assessed is all-cause mortality, stratified by severity of disease at time of randomization. Major secondary outcomes are duration of hospital stay and time to ventilation or intensive care. Trial enrollment was from March 2020 to November 2021, with only RDV continued through the end of enrollment and with inability to show efficacy of the other agents. Overall, 4,146 subjects were assigned to RDV. Interim results did not show a benefit of RDV administration. The Canadian Treatments for COVID-19 trial (CATCO) reported in January 2022 a lower mortality in RDV patients across 52 Canadian hospitals participating in the SOLIDARITY trial (21). Final SOLIDARITY results, released May 2022 and including the last trial phase where the only drug randomized was RDV, showed a small decrease in mortality among adults requiring oxygen but not ventilated (RDV 14.6% vs. control 16.3%; RR 0.87 [95% CI 0.76–0.99], *p* = 0.04) and lower progression to mechanical ventilation and / or death (23.7% vs. 27.1%; RR 0.83 [0.75–0.93], *p* = 0.001), in RDV versus non-RDV local standard of care control subjects. DisCoVeRy study group, funded by the European Union Commission and others was a Phase 3 RCT open-label intention-to-treat population study across 48 sites in the E.U. with primary outcome being the day-15 clinical status measured by the WHO seven-point ordinal scale. The findings, released in February 2022 (22), report no clinical benefit from the use of RDV for inpatients symptomatic for more than 7 days, and who required oxygen support.

Real world experience / retrospective observational studies, non-Gilead miscellaneous funding: Multiple real world efficacy studies of RDV have been published to date (23–36) (Supplementary Table 1). Overall, clinical trial consensus at this time seemed to be that RDV has some effect in shortening time to clinical improvement (24, 37), shortening duration of admission, preventing progression to mechanical ventilation, and some effect in reducing mortality. Although often qualified as mild, the optimal effect of RDV seems to be achieved when drug is provided at an early phase of the disease, e.g., at time of milder clinical severity with benefit driven by patients on no or low-flow oxygen, with full dose completion. Benefits appear most likely when used in combination with steroids, though RDV benefits appear to be driven at least in part by RDV itself and not by concomitant anti-inflammatory therapies (37). Additional review and meta-analysis were provided sponsored by the American College and Physicians Practice Points (38).

This study aimed to compare clinical outcomes between patients who received RDV and those who did not in the population of greater urban NOLA, a region with some of the worst pre-COVID health outcomes in the US and an early epicenter for severe COVID.

## Materials and methods

### Study design and population

In this retrospective study, data were collected from electronic health records, in MediTech at Tulane Medical Center, TMC, and EPIC at University Medical Center, UMC, two academic medical centers located in New Orleans, Louisiana. The data were collected prospectively from March 2020 to September 2021, from adult patients aged ≥ 18 admitted to inpatient care within 7 days of the first COVID-19 diagnosis. The target variable is RDV treatment vs. non treatment. Patients who started RDV before inpatient admission or > 3 days after inpatient admission were not included in the analysis. Whether a patient was started immediately on RDV or as late as 3 days following admission was up to provider discretion. Patients started on RDV more than 3 days following admission were excluded because they would have been likely to be matched to patients healthy enough to be discharged shortly after the index day, thus not qualifying as candidates for RDV treatment. All patients in the study were inpatients, with RDV recommended for 5-day therapeutic course (vs. 3-day preventive course for outpatients). Whether a full 5-day course was administered, or the patient was discharged prior to 5 doses (due to clinical improvement) was based on provider discretion, as was standard of care during the study timeframe. Since this study is retrospective and population-based (for the two institutions involved, from 3/2020 to 9/2021, following the natural progression of the pandemic), there was no prior determination of sample size. All patients admitted to inpatient care on the day of first COVID-19 diagnosis and starting remdesivir within 3 days after inpatient admission were included in the crude analysis. This resulted in a sample size of 448 participants. The prep-to-research queries, listed in Supplementary Table 3, included demographics, health conditions and diseases, diagnoses, medical interventions, medications administered, laboratory results, vital signs, inpatient admission information, and mortality information. When available, data were collected from 6 months prior to the patient’s COVID-19 diagnosis to 9 months thereafter, allowing estimation of baseline health conditions, calculation of the Charlson comorbidity index and information on vital status post COVID-19 diagnosis. Data were stored in the PCORnet Common Data Model format, version 6.0 (2018) (39).

### Endpoints

The primary endpoint was death from any cause within 28 days after inpatient admission. Secondary endpoints were death from any cause within 14 days after inpatient admission and inpatient discharge within 14 days and within 28 days after admission.

### Matching and statistical analyses

Each endpoint was analyzed both in all patients (crude analysis) and in matched patients (matched sample analysis). Exact matching was performed on location of inpatient admission (University Medical Center; Tulane Medical Center) and on calendar period of inpatient admission [March-May 2020 (predominant circulation of the alpha variant, first wave/peak of mortality); June–August 2020 (alpha variant, second wave); September 2020-February 2021 (alpha variant third wave); March-May 2021 (last period of alpha variant predominance); June–September 2021 (predominant circulation of the delta variant)], followed by propensity score matching based on 22 potentially prognostically important baseline covariates listed in Supplementary Table 4. Covariates include select demographics, pre-COVID medical conditions, and clinical status/treatment within 5 days prior to RDV (or corresponding day in patients who did not receive RDV). The matching was performed 1:1 with replacement using the greedy algorithm and a caliper distance of 0.2 of the standard deviation of the logit of the propensity score. To minimize the number of non-RDV patients being matched to ≥ 2 RDV-patients, the following selection process was used: (1) for each RDV-patient, select the non-RDV patient with the smallest caliper distance; (2) if this patient was previously selected for matching, select the patient with the next smallest caliper distance; if all patients within the caliper distance were previously selected for matching, select the non-RDV patient with the smallest caliper distance. Each RDV patient was matched to only one non-RDV patient. However, matching was performed with replacement, i.e., a non-RDV patient could be matched to more than one RDV patient. This is because if matching was performed without replacement, many RDV patients would not have been successfully matched to a non-RDV patient and thus would have been excluded from the analysis. To prevent immortal time bias, a non-RDV patient could be matched to an RDV patient only if the non-RDV patient was still in inpatient care on the index day, defined as the day when the RDV patient initiated remdesivir relative to admission day. The survival time outcome is measured from patient admission, so time prior to patient admission is not included in days of survival outcome. Related to actions preceding admission, all health-related covariates used for the propensity score matching took into account the period preceding admission: (1) All components of the Charlson comorbidity index were assessed “based on past or ongoing medical conditions at baseline,” (2) All other health-related covariates (oxygenation level, pneumonia, sepsis, obesity, concomitant medications) were assessed “on or within 5 days prior to day of RDV initiation or corresponding day in patients who did not receive RDV.” Therefore, they cover at least the 2 days preceding admission (if RDV was initiated 3 days after admission) and at most the 5 days preceding admission (if RDV was initiated on the day of admission). Adequacy of the matching was assessed by standardized differences. Cumulative probabilities of death from any cause within 28 days and within 14 days after inpatient admission—including deaths occurring after discharge—were estimated using the Kaplan-Meier method. In the crude analysis, the association between RDV exposure and death from any cause was assessed using standard Cox proportional hazards regression models. In the matched sample analysis, it was assessed using Cox proportional hazards regression models with shared frailty and a robust sandwich-type variance estimator to account for clustering within matched pairs, clustering within participants and clustering in the cross-classification of these two types of clusters (40, 41). In the matched sample analysis, the Cox proportional hazards regression models were adjusted for all baseline covariates with an absolute standardized difference ≥ 0.15 in at least one category. For all survival analyses, patients discharged before the cutoff day (Day 14 or Day 28) were censored on the cutoff day if they had available medical records after the cutoff day, otherwise on the discharge day. The proportional hazards assumption of each Cox model was assessed graphically using a log-log plot The associations between exposure to RDV and inpatient discharge within 14 days and within 28 days after admission were assessed using Fisher’s exact test in the crude analysis and McNemar’s test in the matched sample analysis. Of note, McNemar’s test does not account for matching with replacement, thus *p*-values should be interpreted with caution. The matching and statistical analyses were performed using Stata version 16.1 (Stata Corp., College Station, TX) and R version 4.3.0.

## Results

### Study population

A total of 448 patients who received RDV and 1,438 patients who did not receive RDV were included in the crude analysis and considered for inclusion in the matched sample analysis (Figure 1). Inpatient admission dates ranged from 12 March 2020 to 20 September 2021. Baseline characteristics of the study population in original and matched samples (Table 1) and in matched and unmatched samples (Table 2) are presented. In the original sample, 1,029 (55%) patients were male, 1,074 (57%) were Black (African American), and the median age was 57 years (interquartile range [IQR]: 43 to 68). The median of the Charlson comorbidity index was 4 (IQR: 2 to 6). Within 5 days prior to the index day, 297 (16%) patients were intubated, and 664 (35%) had respiratory failure, hypoxemia, or dependence on oxygen (Table 1). The study ratio of RDV/non-RDV patients is reported over time (Figure 2). To provide the context in which the ratio evolved, the main milestones relevant to RDV use, pre- and post- RDV FDA EUA, are visually reported in Figure 2 including the release of major randomized clinical trials conclusions, guidelines for the use of RDV in COVID-19 of IDSA, WHO, and FDA. To provide context, official state population data for death related to COVID-19 by variant waves and rate of vaccination is provided in the same graph (Figure 2). The rapid increase of RDV administration reached a 65% peak ∼10 months after FDA EUA, over the 3rd alpha wave and stabilized to ∼50% over the delta wave (Figure 2 orange curve). RDV was more frequently administered to patients with obesity (standardized difference: 0.35) (Table 1). Within 5 days prior to index day, the concomitant administration of steroids was more frequent (standardized difference: 0.88), and patients more likely to receive RDV if experienced pneumonia (0.75), respiratory failure, hypoxemia or were on supplemental oxygen but not intubated (0.69) (Table 1). Of the 448 RDV-patients, 419 (94%) were successfully matched to a non-RDV patient. Of the 1,438 non-RDV patients, 220 were matched to at least one patient who received RDV: 145 (66%) were matched to one RDV patient, 40 (18%) to two, 6 (3%) to three and 29 (13%) to more than three (up to 13). The median absolute standardized difference in the matched sample was 0.06 (IQR: 0.01 to 0.11). Of the 419 RDV patients, 145 (35%) received RDV for less than 5 days, 235 (56%) for 5 days, and 39 (9%) for more than 5 days (Table 3). Steroids were concomitant prescribed within 7 days of admission to 401/419 (96%) RDV-patients and 366/419 (87%) non-RDV patients (Table 3).

**TABLE 1:** Baseline characteristics of the study population in the original and matched samples.

	Original sample			Matched sample		
	Received remdesivir (*N* = 448) *n* (%)	Did not receive remdesivir (*N* = 1,438) *n* (%)	Standardized difference	Received remdesivir (*N* = 419) *n* (%)	Did not receive remdesivir (*N* = 419) *n* (%)	Standardized difference
**Inpatient admission** **Location of inpatient admission**						
University Medical Center	356 (79%)	1,019 (71%)	0.20	338 (81%)	338 (81%)	0.00
Tulane Medical Center	92 (21%)	419 (29%)	−0.20	81 (19%)	81 (19%)	0.00
**Calendar period of inpatient admission**						
March–May 2020	7 (2%)	526 (37%)	−1.00	7 (2%)	7 (2%)	0.00
June–August 2020	57 (13%)	247 (17%)	−0.13	54 (13%)	54 (13%)	0.00
September 2020–February 2021	200 (45%)	329 (23%)	0.47	190 (45%)	190 (45%)	0.00
March–May 2021	32 (7%)	74 (5%)	0.08	22 (5%)	22 (5%)	0.00
June–September 2021	152 (34%)	262 (18%)	0.36	146 (35%)	146 (35%)	0.00
**Demographics**						
Male sex	257 (57%)	772 (54%)	0.07	245 (58%)	264 (63%)	−0.09
Age ≥ 65 years	136 (30%)	479 (33%)	−0.06	130 (31%)	128 (31%)	0.01
**Race**						
Black or African American	229 (51%)	845 (59%)	−0.15	213 (51%)	208 (50%)	0.02
White	123 (27%)	367 (26%)	0.04	118 (28%)	152 (36%)	−0.17
Other/unknown/missing/refused to answer	96 (21%)	226 (16%)	0.15	88 (21%)	59 (14%)	0.18
**Ethnicity**						
Hispanic or Latino	63 (14%)	124 (9%)	0.17	60 (14%)	46 (11%)	0.10
Not Hispanic or Latino	366 (82%)	1,246 (87%)	−0.14	342 (82%)	358 (85%)	−0.10
Other/unknown/missing/refused to answer	19 (4%)	68 (5%)	−0.02	17 (4%)	15 (4%)	0.02
**Primary payer for COVID-19 admission**						
Medicare	153 (34%)	578 (40%)	−0.13	146 (35%)	158 (38%)	−0.06
Medicaid/government/charity/self-pay	185 (41%)	496 (34%)	0.14	171 (41%)	173 (41%)	−0.01
Commercial	66 (15%)	176 (12%)	0.07	63 (15%)	55 (13%)	0.05
Other/missing	44 (10%)	188 (13%)	−0.10	39 (9%)	33 (8%)	0.05
**Location of residence**						
New Orleans, Baton Rouge, Hammond, Jackson or Lafayette	141 (31%)	639 (44%)	−0.27	134 (32%)	107 (26%)	0.14
Other	198 (44%)	618 (43%)	0.02	193 (46%)	172 (41%)	0.10
Missing	109 (24%)	181 (13%)	0.31	92 (22%)	140 (33%)	−0.26
Charlson comorbidity index and components* **at baseline**						
Charlson comorbidity index score ≥ 5	159 (35%)	649 (45%)	−0.20	154 (37%)	174 (42%)	−0.10
Congestive heart failure, myocardial infarction, peripheral vascular disease or cerebrovascular disease*	105 (23%)	429 (30%)	−0.15	100 (24%)	104 (25%)	−0.02
Chronic pulmonary disease*	124 (28%)	371 (26%)	0.04	119 (28%)	125 (30%)	−0.03
Diabetes*	178 (40%)	554 (39%)	0.02	167 (40%)	190 (45%)	−0.11
Renal disease*	236 (53%)	744 (52%)	0.02	225 (54%)	227 (54%)	−0.01
Liver disease or peptic ulcer disease*	31 (7%)	172 (12%)	−0.17	31 (7%)	33 (8%)	−0.02
HIV/AIDS*	6 (1%)	27 (2%)	−0.04	6 (1%)	19 (5%)	−0.18
Lymphoma, leukemia or solid tumor*	34 (8%)	172 (12%)	−0.15	33 (8%)	32 (8%)	0.01
**Dementia**	11 (2%)	138 (10%)	−0.30	11 (3%)	14 (3%)	−0.04
Oxygenation level on or within 5 days prior to day of RDV initiation or corresponding day in patients who did not receive RDV*								
No intubation, respiratory failure, hypoxemia or dependence on supplemental oxygen	98 (22%)	827 (58%)	−0.78	93 (22%)	107 (26%)	−0.08
No intubation but respiratory failure, hypoxemia or dependence on supplemental oxygen	268 (60%)	396 (28%)	0.69	230 (55%)	191 (46%)	0.19
Intubation	82 (18%)	215 (15%)	0.09	96 (23%)	121 (29%)	−0.14
Other indicators of COVID-19 severity or conditions on or within 5 days prior to day of RDV initiation or corresponding day in patients who did not receive RDV*								
Pneumonia	348 (78%)	621 (43%)	0.75	324 (77%)	317 (76%)	0.04
Sepsis	97 (22%)	363 (25%)	−0.08	96 (23%)	70 (17%)	0.16
Obesity	252 (56%)	563 (39%)	0.35	169 (40%)	148 (35%)	0.10
Concomitant medications on or within 5 days prior to day of RDV initiation or corresponding day in patients who did not receive RDV*								
Steroids	259 (58%)	270 (19%)	0.88	359 (86%)	343 (82%)	0.10
Anticoagulants	249 (56%)	764 (53%)	0.05	371 (89%)	321 (77%)	0.32
Monoclonal antibody	16 (4%)	18 (1%)	0.15	17 (4%)	27 (6%)	−0.11

*On or within 5 days prior to day of admission in patients in the original sample who did not receive remdesivir.

**TABLE 2:** Baseline characteristics of the population in the matched and unmatched samples.

	Received remdesivir (*N* = 448)			Did not receive remdesivir (*N* = 1,438)		
	Matched sample (*N* = 419) *n* (%)	Unmatched sample (*N* = 29) *n* (%)	Standardized difference	Matched sample (*N* = 220) *n* (%)	Unmatched sample (*N* = 1,218) *n* (%)	Standardized difference
**Inpatient admission** **Location of inpatient admission**						
University Medical Center	338 (81%)	18 (62%)	0.42	157 (71%)	862 (71%)	0.01
Tulane Medical Center	81 (19%)	11 (38%)	−0.42	63 (29%)	356 (29%)	−0.01
**Calendar period of inpatient admission**						
March–May 2020	7 (2%)	0 (0%)	0.18	7 (3%)	519 (43%)	−1.06
June–August 2020	54 (13%)	3 (10%)	0.08	39 (18%)	208 (17%)	0.02
September 2020–February 2021	190 (45%)	10 (34%)	0.22	85 (39%)	244 (20%)	0.42
March–May 2021	22 (5%)	10 (34%)	−0.79	17 (8%)	57 (5%)	0.13
June–September 2021	146 (35%)	6 (21%)	0.32	72 (33%)	190 (16%)	0.41
**Demographics**						
Male sex	245 (58%)	12 (41%)	0.35	125 (57%)	647 (53%)	0.07
Age ≥ 65 years	130 (31%)	6 (21%)	0.24	66 (30%)	413 (34%)	−0.08
**Race**						
Black or African American	213 (51%)	16 (55%)	−0.09	113 (51%)	732 (60%)	−0.18
White	118 (28%)	5 (17%)	0.26	74 (34%)	293 (24%)	0.21
Other/unknown/missing/refused to answer	88 (21%)	8 (28%)	−0.15	33 (15%)	193 (16%)	−0.02
**Ethnicity**						
Hispanic or Latino	60 (14%)	3 (10%)	0.12	21 (10%)	103 (8%)	0.04
Not Hispanic or Latino	342 (82%)	24 (83%)	−0.03	191 (87%)	1,055 (87%)	0.01
Other/unknown/missing/refused to answer	17 (4%)	2 (7%)	−0.13	8 (4%)	60 (5%)	−0.06
**Primary payer for COVID-19 admission**						
Medicare	146 (35%)	7 (24%)	0.24	86 (39%)	492 (40%)	−0.03
Medicaid/government/charity/self-pay	171 (41%)	14 (48%)	−0.15	79 (36%)	417 (34%)	0.04
Commercial	63 (15%)	3 (10%)	0.14	30 (14%)	146 (12%)	0.05
Other/missing	39 (9%)	5 (17%)	−0.24	25 (11%)	163 (13%)	−0.06
**Location of residence**						
New Orleans, Baton Rouge, Hammond, Jackson or Lafayette	134 (32%)	7 (24%)	0.18	60 (27%)	579 (48%)	−0.43
Other	193 (46%)	5 (17%)	0.65	96 (44%)	522 (43%)	0.02
Missing	92 (22%)	17 (59%)	−0.81	64 (29%)	117 (10%)	0.51
Charlson comorbidity index and components* **at baseline**						
Charlson comorbidity index score ≥ 5	154 (37%)	5 (17%)	0.45	93 (42%)	556 (46%)	−0.07
Congestive heart failure, myocardial infarction, peripheral vascular disease or cerebrovascular disease*	100 (24%)	5 (17%)	0.16	62 (28%)	367 (30%)	−0.04
Chronic pulmonary disease*	119 (28%)	5 (17%)	0.27	66 (30%)	305 (25%)	0.11
Diabetes*	167 (40%)	11 (38%)	0.04	95 (43%)	459 (38%)	0.11
Renal disease*	225 (54%)	11 (38%)	0.32	108 (49%)	636 (52%)	−0.06
Liver disease or peptic ulcer disease*	31 (7%)	0 (0%)	0.40	23 (10%)	149 (12%)	−0.06
HIV/AIDS*	6 (1%)	0 (0%)	0.17	5 (2%)	22 (2%)	0.03
Lymphoma, leukemia or solid tumor*	33 (8%)	1 (3%)	0.19	23 (10%)	149 (12%)	−0.06
Dementia	11 (3%)	0 (0%)	0.23	12 (5%)	126 (10%)	−0.18
**Oxygenation level on or within 5 days prior to day of RDV initiation or corresponding day in patients who did not receive RDV**								
No intubation, respiratory failure, hypoxemia or dependence on supplemental oxygen	95 (23%)	3 (10%)	0.34	86 (39%)	741 (61%)	−0.45
No intubation but respiratory failure, hypoxemia or dependence on supplemental oxygen	244 (58%)	24 (83%)	−0.56	83 (38%)	313 (26%)	0.26
Intubation	80 (19%)	2 (7%)	0.37	51 (23%)	164 (13%)	0.25
**Other indicators of COVID-19 severity or conditions on or within 5 days prior to day of RDV initiation or corresponding day in patients who did not receive RDV**								
Pneumonia	323 (77%)	25 (86%)	−0.24	138 (63%)	483 (40%)	0.47
Sepsis	95 (23%)	2 (7%)	0.46	45 (20%)	318 (26%)	−0.13
Obesity	234 (56%)	18 (62%)	−0.13	101 (46%)	462 (38%)	0.16
**Concomitant medications on or within 5 days prior to day of RDV initiation or corresponding day in patients who did not receive RDV**								
Steroids	237 (57%)	22 (76%)	−0.42	116 (53%)	154 (13%)	0.95
Anticoagulants	233 (56%)	16 (55%)	0.01	119 (54%)	645 (53%)	0.02
Monoclonal antibody	13 (3%)	3 (10%)	−0.29	9 (4%)	9 (1%)	0.22

*Indicates that these components e.g., HIV/AIDS were part of the Charlson’s comorbidity index.

**TABLE 3:** Exposure to remdesivir and concomitant steroids in the original and matched samples.

	Original sample		Matched sample	
	Received remdesivir (*N* = 448) *n* (%)	Did not receive remdesivir (*N* = 1,438) *n* (%)	Received remdesivir (*N* = 419) *n* (%)	Did not receive remdesivir (*N* = 419) *n* (%)
**Day of RDV initiation or corresponding day in patients who did not receive RDV**				
Day of inpatient admission	173 (39%)	–	157 (37%)	157 (37%)
1 day after inpatient admission	218 (49%)	–	208 (50%)	208 (50%)
2 days after inpatient admission	40 (9%)	–	38 (9%)	38 (9%)
3 days after inpatient admission	17 (4%)	–	16 (4%)	16 (4%)
**Duration of exposure to RDV**				
1 day	37 (8%)	–	37 (9%)	–
2 days	21 (5%)	–	20 (5%)	–
3 days	36 (8%)	–	33 (8%)	–
4 days	61 (14%)	–	55 (13%)	–
5 days	250 (56%)	–	235 (56%)	–
6 days	23 (5%)	–	20 (5%)	–
7 days	1 (< 1%)	–	1 (< 1%)	–
8 days	2 (< 1%)	–	2 (< 1%)	–
9 days	1 (< 1%)	–	1 (< 1%)	–
10 days	13 (3%)	–	13 (3%)	–
More than 10 days	3 (1%)	–	2 (< 1%)	–
Received steroids during exposure to RDV	425 (95%)	–	396 (95%)	–
Received steroids within 7 days after inpatient admission	430 (96%)	519 (36%)	401 (96%)	366 (87%)

### Patient outcome over time

**FIGURE 3:**
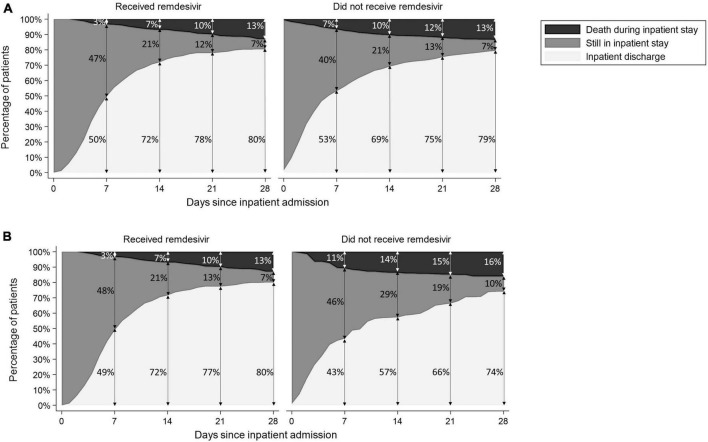
Patient outcome over time in **(A)** crude analysis and **(B)** matched sample analysis.

The proportions of patients discharged, still admitted, and who died during inpatient stay are described over time in Figure 3 in (a) crude and (b) matched sample analysis.

### Death from any cause within 14 and 28 days of admission

**FIGURE 4:**
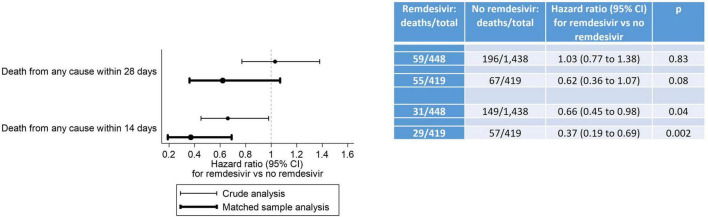
Death from any cause within 28 days and within 14 days after inpatient admission. In the crude analysis, the association between RDV exposure and death from any cause was assessed using standard Cox proportional hazards regression models. In the matched sample analysis, it was assessed using Cox proportional hazards regression models with shared frailty and a robust sandwich-type variance estimator to account for clustering within matched pairs, clustering within participants and clustering in the cross-classification of these two types of clusters. The proportional hazards assumption of the Cox models was met for the 14-day crude and matched sample analyses, but not for the 28-day crude and matched sample analyses. Although studies encountering non-proportional hazards are common, effectively addressing non-proportional hazards remains an ongoing research topic. Despite the proportional hazards assumption not being met for our 28-day analyses, we are following the recommendation from the FDA-initiated “Non-Proportional Hazards Cross-Pharma Working Group” to report hazard ratio estimates from these models: “*In terms of summarizing treatment effect, researchers should continue to present Kaplan-Meier curves and Cox model hazard ratio estimates*” (45).

**FIGURE 5:**
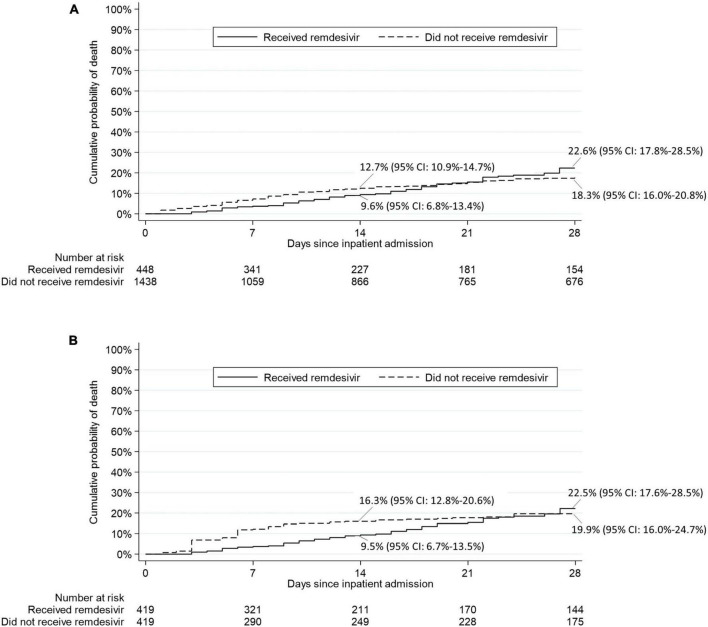
Kaplan-Meier estimates of the cumulative probability of death from any cause within 28 days after inpatient admission in **(A)** crude analysis and **(B)** matched sample analysis.

Results for mortality endpoints from the Cox proportional hazard regression models are provided in Figure 4. In the matched sample analysis, patients who received RDV were significantly less likely to die within 14 days after inpatient admission (hazard ratio [HR]: 0.37, 95% CI: 0.19 to 0.69, *p* = 0.002) but not within 28 days after inpatient admission (HR: 0.62, 95% CI: 0.36 to 1.07, *p* = 0.08) after adjustment for the six baseline covariates with an absolute standardized difference ≥ 0.15 in at least one category (race, location of residence, HIV/AIDS, oxygenation level, sepsis and anticoagulants). The results of the subgroup analysis of death from any cause within 28 days after inpatient admission are provided in Table 4. In the matched sample analysis, exposure to RDV was significantly associated with a lower risk of death in patients admitted between March and May 2021 (HR: 0.11, 95% CI: 0.01 to 0.95, *p* = 0.04) or with no sepsis on or within 5 days prior to index day (HR: 0.39, 95% CI: 0.17 to 0.93, *p* = 0.03). In the matched sample analysis, co-administration of RDV and steroids was not associated with the risk of death (Table 4). Kaplan-Meier estimates of the cumulative probability of death from any cause within 28 days after admission, in crude and matched samples, are provided in Figure 5.

**TABLE 4:** Death from any cause within 28 days after inpatient admission in each stratum of the baseline covariates used for the propensity score matching.

	Remdesivir			No remdesivir				
	Group size	Deaths	28-day cumulative probability of death	Group size	Deaths	28-day cumulative probability of death	Hazard ratio (95% CI) for remdesivir vs. no remdesivir	*p*
Overall	419	55	22.5%	419	67	20.0%	0.62 (0.36–1.07)	0.08
**Inpatient admission** **Location of inpatient admission**								
University Medical Center	338	50	25.0%	338	53	19.0%	1.00 (0.53–1.89)	0.99
Tulane Medical Center	81	5	11.7%	81	14	24.5%	0.33 (0.10–1.06)	0.06
**Calendar period of inpatient admission**								
March–May 2020	7	0	0%	7	0	0%	–	–
June–August 2020	54	15	38.4%	54	3	8.5%	3.96 (0.79–19.73)	0.09
September 2020–February 2021	190	24	19.4%	190	21	12.3%	1.19 (0.40–3.56)	0.75
March–May 2021	22	1	8.3%	22	8	53.4%	0.11 (0.01–0.95)	0.04
June–September 2021	146	15	24.6%	146	35	30.7%	0.46 (0.20–1.08)	0.07
**Demographics** **Sex**								
Male	245	33	23.6%	264	42	19.7%	0.87 (0.40–1.87)	0.72
Female	174	22	21.0%	155	25	20.5%	0.82 (0.38–1.75)	0.61
**Age**								
< 65 years	289	24	14.3%	291	39	17.7%	0.61 (0.29–1.32)	0.21
≥ 65 years	130	31	40.1%	128	28	24.6%	1.24 (0.53–2.88)	0.62
**Race**								
Black or African American	213	30	22.6%	208	41	22.7%	0.75 (0.38–1.51)	0.42
White	118	18	27.2%	152	13	11.9%	2.20 (0.90–5.41)	0.08
Other/unknown/missing/refused to answer	88	7	16.1%	59	13	30.6%	0.25 (0.06–1.02)	0.05
**Ethnicity**								
Hispanic or Latino	60	10	27.7%	46	9	22.0%	0.68 (0.12–3.91)	0.66
Not Hispanic or Latino	342	44	21.6%	358	57	19.7%	0.87 (0.49–1.52)	0.62
Other/unknown/missing/refused to answer	17	1	20.0%	15	1	11.1%	0.95 (0.02–57.46)	0.98
**Primary payer for COVID-19 admission**								
Medicare	146	30	34.9%	158	31	24.1%	1.10 (0.52–2.34)	0.80
Medicaid/government/charity/self-pay	171	18	16.6%	173	27	17.6%	0.69 (0.25–1.86)	0.46
Commercial	63	5	14.1%	55	5	14.5%	1.05 (0.27–4.11)	0.94
Other/missing	39	2	13.4%	33	4	32.7%	0.29 (0.05–1.84)	0.19
**Location of residence**								
New Orleans, Baton Rouge, Hammond, Jackson or Lafayette	134	13	16.1%	107	8	9.1%	1.53 (0.54–4.33)	0.42
Other	193	26	26.4%	172	40	29.0%	0.63 (0.28–1.41)	0.26
Missing	92	16	24.9%	140	19	17.1%	1.14 (0.40–3.20)	0.81
Charlson comorbidity index and components* **at baseline** **Charlson comorbidity index score**								
< 5	265	22	15.1%	245	34	17.4%	0.63 (0.26–1.53)	0.31
≥ 5	154	33	33.2%	174	33	23.3%	1.12 (0.57–2.20)	0.73
Congestive heart failure, myocardial infarction, peripheral vascular disease or cerebrovascular disease*								
Yes	100	22	32.4%	104	21	24.3%	1.07 (0.49–2.36)	0.86
No	319	33	19.1%	315	46	18.4%	0.75 (0.36–1.57)	0.44
Chronic pulmonary disease*								
Yes	119	27	33.1%	125	18	16.8%	1.64 (0.68–3.99)	0.27
No	300	28	17.0%	294	49	21.6%	0.57 (0.28–1.16)	0.12
Diabetes*								
Yes	167	26	24.8%	190	28	18.0%	1.13 (0.55–2.30)	0.74
No	252	29	20.6%	229	39	21.5%	0.67 (0.30–1.46)	0.31
Renal disease*								
Yes	225	33	23.6%	227	48	24.9%	0.66 (0.33–1.33)	0.25
No	194	22	21.0%	192	19	14.1%	1.36 (0.57–3.23)	0.49
Liver disease or peptic ulcer disease*								
Yes	31	7	30.2%	33	12	45.7%	0.44 (0.14–1.45)	0.18
No	388	48	21.6%	386	55	17.8%	0.93 (0.50–1.72)	0.81
HIV/AIDS*								
Yes	6	1	33.3%	19	0	0%	–	–
No	413	54	22.3%	400	67	20.6%	0.81 (0.47–1.42)	0.47
Lymphoma, leukemia or solid tumor*								
Yes	33	8	30.4%	32	3	11.7%	2.81 (0.69–11.47)	0.15
No	386	47	21.8%	387	64	20.6%	0.76 (0.42–1.36)	0.35
**Dementia**								
Yes	11	2	25.9%	14	2	18.0%	1.41 (0.18–11.15)	0.75
No	408	53	22.2%	405	65	20.0%	0.84 (0.48–1.47)	0.54
**Oxygenation level on or within 5 days prior to day of RDV initiation or corresponding day in patients who did not receive RDV**								
No intubation, respiratory failure, hypoxemia or dependence on supplemental oxygen	93	4	7.7%	107	2	3.1%	2.29 (0.38–13.87)	0.37
No intubation but respiratory failure, hypoxemia or dependence on supplemental oxygen	230	22	18.7%	191	15	9.9%	1.48 (0.51–4.27)	0.47
Intubation	96	29	41.0%	121	50	45.5%	0.64 (0.33–1.23)	0.18
**Other indicators of COVID-19 severity or conditions on or within 5 days prior to day of RDV initiation or corresponding day in patients who did not receive RDV**								
**Pneumonia**								
Yes	324	50	26.8%	317	60	22.9%	0.87 (0.47–1.61)	0.65
No	95	5	9.3%	102	7	9.5%	0.73 (0.20–2.62)	0.63
**Sepsis**								
Yes	96	41	52.0%	70	28	43.7%	0.98 (0.50–1.94)	0.96
No	323	14	8.2%	349	39	14.2%	0.39 (0.17–0.93)	0.03
**Obesity**								
Yes	169	21	19.9%	148	31	25.3%	0.65 (0.30–1.41)	0.28
No	250	34	24.4%	271	36	16.4%	1.03 (0.47–2.27)	0.93
**Concomitant medications on or within 5 days prior to day of RDV initiation or corresponding day in patients who did not receive RDV**								
**Steroids**								
Yes	359	48	23.2%	343	63	22.2%	0.76 (0.42–1.37)	0.36
No	60	7	18.6%	76	4	8.4%	2.10 (0.59–7.49)	0.25
**Anticoagulants**								
Yes	371	52	24.2%	321	62	23.9%	0.72 (0.40–1.29)	0.27
No	48	3	10.0%	98	5	6.7%	1.41 (0.32–6.19)	0.65
**Monoclonal antibody**								
Yes	17	4	28.2%	27	10	43.7%	0.52 (0.11–2.50)	0.41
No	402	51	22.1%	392	57	18.1%	0.91 (0.50–1.66)	0.77

*Indicates that these components e.g., HIV/AIDS were part of the Charlson’s comorbidity index.

### Inpatient discharge

Results for the inpatient discharge endpoints are provided in Table 5. In the matched sample analysis, patients who received RDV were significantly more likely to be discharged within 14 days after admission (*p* < 0.001) and numerically more likely to be discharged within 28 days after admission (*p* = 0.06).

**TABLE 5:** Inpatient discharge within 14 days and within 28 days after admission.

	Crude analysis			Matched sample analysis		
	Received remdesivir (*N* = 448) *n* (%)	Did not receive remdesivir (*N* = 1,438) *n* (%)	*p* *	Received remdesivir (*N* = 419) *n* (%)	Did not receive remdesivir (*N* = 419) *n* (%)	*p* **
Inpatient discharge within 14 days after admission	322 (72%)	994 (69%)	0.29	300 (72%)	240 (57%)	< 0.001
Inpatient discharge within 28 days after admission	360 (80%)	1,141 (79%)	0.69	335 (80%)	310 (74%)	0.06

*From Fisher’s exact test. **From McNemar’s test.

## Discussion

In this study of 419 propensity score-matched adult patients hospitalized with COVID-19 in the NOLA region, patients who received RDV were significantly less likely to die within 14 days after inpatient admission (hazard ratio [HR]: 0.37, 95% CI: 0.19 to 0.69, *p* = 0.002) and numerically—but not significantly—less likely to die within 28 days after inpatient admission (HR: 0.62, 95% CI: 0.36 to 1.07, *p* = 0.08). These findings are consistent with those observed in Beigel et al. (13) by day 15 (HR: 0.55, 95% CI: 0.36 to 0.83) and day 29 (HR: 0.73, 95% CI: 0.52 to 1.03) (13). Interestingly, in the current study exposure to RDV was significantly associated with lower risk of death from any cause within 28 days (Table 4) for patients, 1- who did not experience sepsis (on or within 5 days prior to index day) (HR: 0.39, 95% CI: 0.17 to 0.93, *p* = 0.03) and 2- for patients admitted between the 3rd alpha wave and the delta wave, March to May 2021 (HR: 0.11, 95% CI: 0.01 to 0.95, *p* = 0.04) though *n* was small in this subgroup (*n* = 22 RDV, *n* = 22 no-RDV). RDV was not associated with a significantly lower risk of 28-day mortality in other calendar periods or in any of the baseline COVID-19 severity subgroups. In Beigel et al. (13), the only baseline COVID-19 severity subgroup in which RDV was associated with a lower risk of death by day 29 was observed in patients requiring supplemental oxygen but not requiring ventilation nor the use of high-flow oxygen. IDSA guidelines released June 2021 [v4.3 (#11),] (Figure 2 and Supplementary Table 2) advocated against administration of RDV for patients requiring mechanical ventilation. This potential restriction of RDV benefit to low-flow oxygen patients only was likely reflected in prescribing methods, as the last alpha and the delta waves were coincident with a similar high proportion of RDV/non-RDV patients (64 vs. 54%), suggesting less RDV was administered to patients with severe forms of COVID-19. The overall cumulative probability of death from any cause within 28 days after admission in non-RDV patients was 22.5% in the matched sample analysis. This estimate was much higher than that observed in the control group of all previous RCTs in hospitalized patients with COVID-19: 15.6% in the WHO Solidarity trial, 2% in Spinner et al. (16), 9% in Ader et al. (22), 13% in Wang et al. (11) and 15.2% in Beigel et al. (13) (Supplementary Table 1).

The higher estimate in our study could be explained by the delta variant: all RCTs cited above were conducted before the emergence of the delta variant, and we observed a higher risk of death during delta variant-predominant circulation than during any alpha variant wave. The high cumulative probability of death we observed did not seem to be explained by severity of COVID-19 at baseline, as, for example, baseline proportions of patients intubated and dependent on supplemental oxygen were lower than in Beigel et al. (13) and Ader et al. (22) (Supplementary Table 1). Other potential contributors to the high death rate in our cohort include (1) an overall high rate of sepsis as well as (2) the high pre-COVID morbidity in the NOLA region, among US worst for most health metrics. These two factors could also be related to each other, with pre-COVID comorbidities such as obesity and diabetes increasing the likelihood of peri-COVID complications.

Patients who received RDV were significantly more likely to be discharged within 14 days (72 versus 57%, *p* < 0.001) (Table 4) and only numerically but not significantly more likely within 28 days (80 vs. 74%, *p* = 0.06). The proportion of patients discharged within 14 days among those who did not receive RDV (57%) was higher than that observed in the control group of Wang et al. (11) (23%), Ali et al. (21) (41%), Beigel et al. (13) (42%) and Ader et al. (22) (49%), (Supplementary Table 1) possibly due to the higher proportion of patients with no dependence on supplemental oxygen.

The current study is unique as it focuses upon the effect of RDV administered for COVID-19 among the inhabitants of a single region of the Gulf South (greater urban NOLA), and as it is the only study to cover waves of infection during predominant circulation of the alpha and delta variants of SARS CoV-2 (March 20-Sept. 21, Figure 2). The characteristics of the patients recruited in the study are fully representative of the regional population by reports of the US Census Bureau, Center for Diseases Control (CDC), LA Department of Health, and the Bogalusa Heart Study for age, (68.5% patients < 65 years), race (55% Black), experience of pronounced poverty (37% on Medicaid / not insured), and poor baseline health reflected by high prevalence of pre-COVID-19 chronic health conditions (48% obese, 52.5% chronic kidney disease, 40% diabetes mellitus, 26% chronic heart disease, 27% chronic lung disease and 20% with substance use disorder). The proportion of patients who died during inpatient stay within 28 days from admission in RDV vs. non-RDV was 13 and 14% for crude analysis and 17 and 23% in the matched analysis, similar to the initial RCT with RDV (11), identical to the RDV compassionate use study (12), and only slightly above that of the ACTT-1 trial (13).

This study is a testimony of the clinical practice in the NOLA urban region over the pandemic. It is worth mentioning that, in contrast to several other US regions and multiple other countries (42), LA did not experience shortage of oxygen. Each patient admitted was provided oxygen supplement according to SOC, upon clinical evaluation, hemoglobin oxygen saturation measurement and with daily monitoring until patient returned to baseline oxygen/no supplemental oxygen requirement. Our clinical practice data provides 5-day RDV effectiveness with, for most patients, co-administration of corticosteroids. The best comparator studies is a report of patient outcomes over the very first months of the COVID-19 epidemic in LA, performed at the Ochsner Medical Center (43), in the same urban setting, with a much higher percent of Black patients admitted (70%), a mortality of 30% in Black vs. 21% in White patients, but no association between Black race and in-hospital mortality. In the Ochsner cohort, however, the percent of patients on Medicaid was lower, the Charlson CI lower, at that time corticosteroids were not recommended as standard practice, and RDV was only available through compassionate use. Comparison with the RWE of Garibaldi et al. (25) is difficult: the multicenter study includes our Tulane Medical Center (TMC) site, but the study design contrasts from the current study which is geographically focused, in a single population applying the same distancing measures, exposed to the circulation of the same dominant variant, with specific timing / proportion of population with vaccination completed over time preventing bias introduced by variables that are not synchronized from one region to another.

The best matched study, as comparator, is Goldman et al. (15), for 80% of the patients of the cohort homogeneous RDV administration rate and ratio were observed. The low death rate reported in our RWE may be attributed to several factors, including the co-administration of RDV with corticosteroids (58% in RDV and 19% in non-RDV for crude and 86 and 82% after match). In contrast, the cumulative probability of death in White patients (27% in RDV and 12% in non-RDV) and in Black patients (23 and 23%) is closer to that of the Ochsner cohort, though over a much longer period in the present study. Unfortunately, other comparator studies are not available, as in the US, clinical trial activity frequently concentrates in bicoastal states, neglecting middle regions including the impoverished Gulf South. The Gulf South should be perceived as a valuable US region of interest for clinical trials due to its high concentration of high social vulnerability. Data on expensive treatments that work in a bicoastal population with relatively intense healthcare access may not necessarily be transposable to the Gulf South population or even the more neglected pockets (e.g., the South Bronx) within well-resourced US regions. Health disparities observed in COVID-19 were reflective of competitive clinical trial access patterns in the US and highlight an opportunity for improvement.

This RWE of RDV effectiveness is also unique in corroborating the overall effectiveness of RDV, here in crude and matched analysis upon survival within 28 days of admission.

Among the 22 co-variates used for PS calculation, the Charlson comorbidity index (CCI) is quite low. Although the cohort’s patients display a high prevalence of co-morbidities associated with risk for severe COVID-19 outcomes, the CCI may not reflect optimally the extent to which poor patients of relatively young age may be affected. The CCI is a commonly used index but is limited for prediction of risk in patients with acute COVID-19: the weight attributed to some criteria such as 1- HIV is excessive, outdated, as not reflecting the near cure benefit from Anti Retro Viral (ARV) in multi-therapy, 2-cancers are much less associated with immunosuppression depending upon the type solid vs. hematologic, and time from last therapy, 3- the weight of age is not adapted to our population as accumulation of advanced health issues at young age is not incremented, 4- criteria of major significance such as obesity, substance use disorder, effect of co-morbidities clustering and vaccination status are not included in the CCI. In addition, the comparison from one study to another is difficult as multiple versions of the CCI are used (44).

We observe unique characteristics of RDV prescription in RWE over several waves of the pandemic. First, a rapid increased proportion of inpatients prescribed RDV, with a peak RDV/non-RDV inpatients ratio > 50% by the 3rd alpha wave (September 2020–April 2021) and over the delta wave (July–September 2021) (orange curve, Figure 2). This curve paralleled with LA population vaccination coverage as ∼ 50% of the population had completed full vaccination by July 2021 (green curve, Figure 2). In our clinical experience, however, the actual proportion of hospitalized vaccinated patients was low. Second, RDV was prescribed more if patient was obese, had pneumonia, required supplemental oxygen (but not intubation), and was prescribed steroids within 5 days prior to index day (58 vs. 19%) (Table 1).

The rapid increase of patients (absolute and proportion) who received RDV (Figure 2, orange curve) likely reflects wider availability of the drug after Emergency Use Authorization (EUA) by FDA and increased confidence toward benefit attributed to RDV, especially after ACTT-1 study and FDA final approval in September–October 2020. The graph suggests that the interim WHO guidelines based the SOLIDARITY trial against the use of RDV in COVID-19 released in November 2020, did not stop the prescription of RDV. Both study sites (TMC and UMC NOLA) endorsed the guidance from the EUA for RDV administration: significant COVID-19 illness with SpO2 ≤ 94% on room air or required supplemental oxygen, invasive mechanical ventilation, and alanine aminotransferase (ALT) less than five time the upper limit of normal. However, the decision to prescribe RDV was made by the attending physician for each individual patient. The inflection in the proportion of RDV administration, from 64% at the peak of 3rd alpha wave to 54% at the peak of the delta wave, may be explained by the release of the IDSA guidelines vs. 4.3 in June 2021 suggesting against the administration of RDV in patients with requirement for MV (Figure 2 and Supplementary Table 2, IDSA 4.3 recommendation # 10b).

Our study has several limitations, mostly inherent to the aim of focusing on a specific region’s underrepresented population, driving the number of recruits down. It is possible that scientific and public debates regarding RDV benefit, risk, and cost had an unmeasured influence on provider use of RDV at various points in the pandemic. Specifically, an unscalable possible bias of our study is related to the consequences of the release of the interim SOLIDARITY results upon local physician’s confidence toward RDV administration and benefit. It was noticeable, however, from direct interactions at patients’ bedside, files reviews and professional meetings/interactions, that while most physicians were compliant with national IDSA recommendations, some prioritized WHO guidelines. This dual perception regarding RDV administration may have ultimately optimized the proportion of matching control patients. Lack of control for this influence is a limitation of this study and something that might be anticipated and explored in future public health crises. The vaccination status was not available for most patients in the EMR and the only reportable rate was taken from state statistics. Our study population with higher co-morbidities and low healthcare access had consistently low vaccination coverage on admission (ClinSeqSer cohort, unpublished data).

Strengths of the study include stable oxygen supply, the inclusion of two systems of care, one private and the other a safety net non-profit, public hospital (UMC), that shared standard of care, scheduled consensus meetings, where physicians are rotating in both centers but with dissimilar patients on admission (by primary payer, proportion of homeless population, ZIP codes), while spanning over multiple peaks of death over the waves of alpha variants over the delta variant and in the interim period.

Compared with multicenter studies and larger cohorts, the relative lower number of recruits are compensated by the strengths of focusing the study on a single homogeneous population, in a specific region, over a non-previously reported period extending from the early phase to the delta wave in the summer of 2021. Our study is unique as it shows that RDV has benefit in populations that have been underrepresented in clinical trials and is consistent with observation provided in the study by Garibaldi et al. (25).

## Conclusion

This study suggests that in a cohort representative of the greater urban NOLA region, RDV was associated with a survival benefit, especially in non-sepsis patients, and more likely discharge to home within 14 (albeit not 28) days of admission for acute COVID-19 treatment. This study corroborates the overall findings regarding RDV efficacy, here mostly in association with steroids. The finding that RDV treatment is associated with more likely discharge within 14, but not 28, days, raises the possibility that RDV is protective against early-phase virus-mediated pathology, but not the later-phase inflammatory pathology of COVID. We conclude that use of RDV for COVID-19 is supported by this data, but must be paired with ongoing study of evolving recommendations for late-COVID steroids and other potential immunomodulators. This study is unique for several reasons: 1- its representation of both White and Black patients living in a high state of impoverishment, with advanced pre-COVID-19 health issues and relatively low vaccination coverage, 2-describing RDV effect over the circulation of two major SARS CoV-2 variant circulation periods with deadly outcomes (1st to 3rd alpha waves and the delta wave), and 3- by its illustration of characteristics of RDV prescription in obese subjects, patients with pneumonia and patients with dementia. In conclusion, both small benefit on survival and on admission length are cumulatively valuable and remarkable in this impoverished population where severity of chronic health problems is correlated with expectation of high risks for acute respiratory viral infection and high death rate. Importantly, our real-world experience shows no noticeable inequity in RDV administration as benefits were observed with similar effectiveness of RDV for acute COVID-19 regardless of race, sex, ZIP code, primary payer, or medical center/health admission. This study reflects the remarkable effectiveness and dedication of the healthcare workers over the pandemic in LA, as well as the long road ahead to improved baseline health, across races, in the region.

## Data availability statement

The raw data supporting the conclusions of this article will be made available by the authors, without undue reservation.

## Ethics statement

The studies involving humans were approved by the Tulane Human Research Protections Office (HRPO) and UMC Office of Human Research (Protocol #2021-112) in June 2021 and August 2021, respectively, under the Title “Real World Data Observational Study of COVID-19 in a Flyover Region” [Tulane Human Research Protection Office/IRB Ref. #773437]. The studies were conducted in accordance with the local legislation and institutional requirements. Written informed consent for participation was not required from the participants or the participants’ legal guardians/next of kin in accordance with the national legislation and institutional requirements.

## Author contributions

NS: Conceptualization, Data curation, Formal analysis, Investigation, Methodology, Software, Supervision, Validation, Visualization, Writing – original draft, Writing – review & editing. MF: Data curation, Formal analysis, Investigation, Methodology, Software, Supervision, Validation, Visualization, Writing – original draft, Writing – review & editing. MC: Conceptualization, Formal analysis, Investigation, Methodology, Validation, Visualization, Writing – original draft, Writing – review & editing. LC: Conceptualization, Formal analysis, Investigation, Methodology, Validation, Visualization, Writing – original draft, Writing – review & editing. CW: Conceptualization, Formal analysis, Investigation, Methodology, Validation, Visualization, Writing – original draft, Writing – review & editing. EL: Conceptualization, Formal analysis, Investigation, Methodology, Validation, Visualization, Writing – original draft, Writing – review & editing. VF: Conceptualization, Methodology, Writing – review & editing. DF: Conceptualization, Formal analysis, Investigation, Methodology, Validation, Visualization, Writing – original draft, Writing – review & editing. GJ: Conceptualization, Data curation, Formal analysis, Investigation, Methodology, Supervision, Validation, Visualization, Writing – original draft, Writing – review & editing. AD: Conceptualization, Data curation, Formal analysis, Funding acquisition, Investigation, Methodology, Project administration, Resources, Supervision, Validation, Visualization, Writing – original draft, Writing – review & editing.
