# Supplementary material for: Real-world evidence of survival benefit of remdesivir: study of 419 propensity score-matched patients hospitalized over the alpha and delta waves of COVID-19 in New Orleans, LA
Source: Front Med (Lausanne). 2024 May 16;11:1390164. doi: 10.3389/fmed.2024.1390164 (PMC11137210; doi:10.3389/fmed.2024.1390164)
Supplement: Supplementary file 1 [file Table_1.DOCX]

**Suppl. Table S1: Remdesivir studies, randomized clinical trials and real-world experience trials summary.**

| **1^st^ Author**  **Study acronym**  **sponsor** | **PMID** | **Design** | **Location** | **Calendar time of recruitment** | **Main findings** | **Comments** |
| --- | --- | --- | --- | --- | --- | --- |
| Grein J.  Sponsor  Gilead Sciences, Inc. | 32275812 | 53 patients with severe COVID-19 [ox sat ≤94% on RA or receiving ox sup.]  RDV 10 day course, dose day 1 of 200mg thereafter 100mg daily | Multicenter US, EU, CA, Japan | 1.25 to 3. 07.20 | clinical improvement was observed in 36 of 53 patients (68%) | Non randomized non placebo controlled |
|  |  | **RANDOMIZED CLINICAL TRIALS** | | | |  |
| Wang  [NCT04257656](https://clinicaltrials.gov/show/NCT04257656)  Sponsor:  Chinese Academy of Medical Sciences Emergency Project of COVID-19 | 32423584 | **Randomized, double-blind, placebo-controlled:** Eligible patients were adults (aged ≥18 years) admitted to hospital with laboratory-confirmed SARS-CoV-2 infection, with an interval from symptom onset to enrolment of 12 days or less, oxygen saturation of 94% or less on room air or a ratio of arterial oxygen partial pressure to fractional inspired oxygen of 300 mm Hg or less, and radiologically confirmed pneumonia. Patients were randomly assigned in a 2:1 ratio to intravenous RDV (200 mg on day 1 followed by 100 mg on days 2–10 in single daily infusions) or the same volume of placebo infusions for 10 days | multicenter trial at ten hospitals in Hubei, China. | 2.6. to 3.12.20 | 237 patients were enrolled and randomly assigned to a treatment group (158 to RDV and 79 to placebo); one patient in the placebo group who withdrew after randomization was not included in the ITT population. RDV use was not associated with a difference in time to clinical improvement (hazard ratio 1·23 [95% CI 0·87–1·75]). | Although not statistically significant, patients receiving RDV had a numerically faster time to clinical improvement than those receiving placebo among patients with symptom duration of 10 days or less (hazard ratio 1·52 [0·95–2·43]). |
| Beigel  ACTT-1  Gilead Sciences, Inc. | 32445440 | Phase 3, randomized **double-blind placebo-controlled**, 541 RDV v 521 placebo, 10 day course | 60 sites & 13 sub-sites:  US (45) Denmark (8), UK (5), Greece (4), Germany (3), Korea (2), Mexico (2), Spain (2), Japan (1), Singapore (1) | 2.21. to 4.19.20 | decreased time to recovery (1-2d) in hospitalized patients on no or low-flow oxygen, but no mortality benefit. Included 1062 inpatients | A critique of this study is the at-enrolment higher proportion of good-prognosis inpatients among RDV compared to non-RDV controls (WHO SOLIDARITY FINAL DATA. Lancet 2022) |
| Olender  SIMPLE-severe  Gilead Sciences, Inc. | 32706859 | compared a prospective Phase 3 study to a retrospective cohort; compared efficacy of RDV vs SOC in adults with COVID-19 using data from both  1) phase 3 RDV trial # 5773 and (5 vs 10 day course)  2) retrospective cohort of severe COVID-19 pts treated with SOC trial # 5807 | **5773-** 45 sites in US, Italy, Spain, Germany, Hong Kong, Singapore, South Korea, Taiwan;  **5807-** 16 sites in US, UK, Belgium, Singapore, S Korea | 5773-  3.9. to 4.10.20  5807-  2.6. to 4.10.20 | Comparison between 312 patients who received RDV and 818 non-RDV patients found that at day 14 RDV-treated patients had greater recovery (74% vs 59%, aOR 2.03, p<0.001) and decreased odds of death at day 14 (7.6% RDV, versus 12.5% non-RDV | is it important that they also mention a D14, but not 28, reduced risk of death.Is there a trend toward an early protection that “wears off”. |
| Spinner  SIMPLE moderate  Sponsor Gilead Sciences, Inc. | 32821939 | **Randomized**, open-label, phase 3 RDV trial (5 vs 10 day course) in  hospitalized patients with confirmed SARS-CoV-2 and moderate COVID-19 pneumonia (pulmonary infiltrates and room-air oxygen saturation >94%) | 105 hospitals in US, Europe, and Asia. | 3.15.20 to 4.18.20 | 584 patients with moderate COVID-19, the day 11 clinical status distribution measured on a 7-point ordinal scale was significantly better for those randomized to a 5-day course of RDV (median length of treatment, 5 days) compared with those randomized to standard care. The difference for those randomized to a 10-day course (median length of treatment, 6 days) compared with standard care was not significantly different. 193 10d RDV, 191 5d RDV, 200 SOC |  |
| Ader DisCoVeRy | 34534511 | Inpatients, phase 3, open-label, adaptive, multicenter, randomized, controlled trial including RDV 5-10 days, conducted at 48 sites in Europe (France, Belgium, Austria, Portugal, Luxembourg). Adult patients (aged ≥18 years) admitted to hospital with laboratory-confirmed SARS-CoV-2 infection and illness of any duration were eligible if they had clinical evidence of hypoxemic pneumonia, or required oxygen supplementation | 48 sites in EU | 3.22.20 to 1.21.21 | 857 participants, no clinical benefit was observed from the use of remdesivir in patients who were admitted to hospital for COVID-19, were symptomatic for more than 7 days, and required oxygen support. |  |
| Ali  CATCO  Sponsor WHO  SOLIDARITY CA | 35045989 | Inpatients, open-label, pragmatic RCT, in conjunction with the Solidarity trial, randomized to 10 days of RDV, plus standard care (SOC), or SOC alone. The primary outcome was in-hospital mortality. | 52 Canadian hospitals | 8.14.20 to 4.01.21 | 1282 RDV patients, in-hospital mortality 18.7% vs. 22.6% in the SOC arm (relative risk [RR] 0.83 (95% confidence interval [CI] 0.67 to 1.03); 60-day mortality 24.8% and 28.2%, respectively (95% CI 0.72 to 1.07); need for mechanical ventilation 8.0% with RDV, and 15.0% in SOC (RR 0.53, 95% CI 0.38 to 0.75). Mean oxygen-free and ventilator-free days at day 28 were 15.9 (± SD 10.5) and 21.4 (± SD 11.3) in RDV and 14.2 (± SD 11) and 19.5 (± SD 12.3) in SOC (*p* = 0.006 & 0.007) | This study raised doubt around the low efficacy of RDV of the overall WHO Solidarity RCT |
| Sponsor WHO  SOLIDARITY interim  Release  NEJM 11.2020 | 33264556 | **randomly** assigned inpatients with Covid-19 equally between one of the trial drug regimens that was locally available and **open control** (up to five options, four active and the local standard of care, RDV regimen was 10 days) | 405 hospitals in 30 countries, from all six WHO regions | 01.3.22. to  10.4 20 | No drug reduced mortality, overall or in any subgroup, or reduced initiation of ventilation or hospitalization duration. 11,330 adults underwent randomization; 2750 were assigned to receive RDV, 954 to hydroxyl-chloroquine, 1411 to lopinavir (without interferon), 2063 to interferon (including 651 to interferon plus lopinavir), and 4088 to no trial drug. | This study raised doubts regarding RDV efficacy. Trial drugs were RDV, HQ, LPV, and IFNb1a, discontinued for futility: HQ June 19 2020, LPV July 4 2020, IFNb1a Oct 16 2020 |
| Sponsor WHO  SOLIDARITY Final  Release  Lancet 5. 2022 | 35512728 | prospective phase 3 **open label** **randomized** study of RDV (up to 10 days) vs non-RDV (not placebo controlled) | hospitalized pts at international sites (35 countries: 31% Europe or Canada, 15% Latin America, 54% Asia and Africa) | 3.22.20 to 1.29.21 | 4146 RDV (10 infusions unless discharged earlier) versus 4129 no study drug. Steroid use was similar between RDV (2782/4146, 67.1%) and non-RDV (2820/4129, 68.3%) groups. Mortality was 14.5% in RDV group, versus 15.6% in non-RDV group [RR 0.91, CI 0.82 to 1.02, p=0.12]. | The WHO conclusion from this study was that hospitalized patients who are not already ventilated experience small benefit against death or progression to ventilation from RDV Solidarity did not find that RDV expedited time to discharge. The final SOLIDARITY publication (Lancet 2022) also included a meta-analysis of all randomized trials, with findings similar to the SOLIDARITY trial mortality data, showing that RDV provides a small mortality benefit to patients not already ventilated. ///palliative discharges were counted as in-hospital deaths, not discharges////. |
| Gottlieb  PINETREE  Sponsor:  Gilead Sciences, Inc. | 34937145 | **OUTPATIENTS, 3 day regimen*:** randomized, **double-blind, placebo-controlled** trial involving non-hospitalized patients with Covid-19 who had symptom onset within previous 7d and had at least one risk factor for disease progression (age ≥60 years, obesity, or certain coexisting medical conditions). Patients were randomly assigned to receive IV RDV (200mg D1, 100mg D2, 3) or placebo | 64 sites in US, Spain, Denmark  and UK | 9.18.20 to 4.8.21 | Among non-hospitalized patients who were at high risk for Covid-19 progression, a 3-day course of RDV had an acceptable safety profile and resulted in an 87% lower risk of hospitalization or death than placebo. A total of 562 patients who underwent randomization and received at least one dose of RDV or placebo were included in the analyses: 279 patients in the RDV group and 283 in the placebo group. The mean age was 50 years, 47.9% of the patients were women, and 41.8% were Hispanic or Latinx. The most common coexisting conditions were diabetes mellitus (61.6%), obesity (55.2%), and hypertension (47.7%) |  |
|  |  | **RETROSPECTIVE AND PROPENSITY SCORE MATCHED STUDIES** | | | |  |
| GARIBALDI | 34910128 | retrospective comparative effectiveness study using time-dependent propensity scores, 5 day RDV course | patients hospitalized for COVID-19 at HCA hospitals in the US; even appendix gives no more info than that | 2.23.20 to 2.11.21 –  ALPHA ONLY  prior to delta | compared 18,328 RDV vs 18,328 non-RDV COVID-19 patients and found that RDV associated with increased likelihood of clinical improvement in patients on no or low-flow oxygen, and decreased mortality among patients on low-flow oxygen | this is HCA study; 54% non-white. In propensity matched group, 83 v 78% dexamethasone for RDV vs controls (SD 0.11). P12 discusses dexamethasone. |
| Frost | 33287853 | observational chart review, comparison to 10 day RDV course (ACTT-1) | Denmark | 3.1. to 5.5.20 | patient characteristics and outcomes in ACTT-1 differ from present real-world population: most pronounced differences are doubled mortality rate and larger proportion of patients only requiring supplemental oxygen in the Danish real-world cohort. Increased mortality rate likely due to cohort's higher age [3]. | purpose- to compare ACTT-1 placebo group to non-RDV Danish cohort ie, is ACTT-1 population generalizable. |
| Flisiak | 33382547 | observational study of 122 RDV (5-10 days) vs. 211 lopin/riton patients | Poland-SARSTer national database-  30 centers | 3.1. to 8.31.20 | logistic regression model of patients receiving RDV or lopinavir / ritonavir, only RDV use independently associated with at least 2-point improvement on ordinal scale between baseline and D21, while older age and tocilizumab use were negative predictors of patient response | lopin/riton comparator group was earlier and more often co admin. chloroquine, RDV more often DXM, convalescent patient plasma, and LMWH |
| Mehta | 33647517 | retrospective chart review looking at impact of sx onset to RDV tx interval on outcome in 346 RDV pts (5-10 days) | India- one tertiary care center | 6.25. to 10.3.20 | RDV initiation <=9d from sx onset aw mortality benefit | 260 <=9d; 86>9d; RDV initiation ≤9 days from symptom onset was associated with mortality benefit, defining a treatment window and reinforcing need for appropriately timed RDV in moderate-to-severe COVID-19. |
| Larson | 35277723 | retrospective analysis of impact of RDV (5-10 days) and dexamethasone on real-world outcomes in severe COVID-19; 44 treated vs 14 untreated historical controls | US, one community hospital (Fort Belvoir) in VA | 3.20 to 12.31.20 | no impact on duration of hospitalization when RDV and dexamethasone added to supportive care in community hospital | study was not sufficiently powered to detect the previously described mortality benefit of dexamethasone |
| Benfield | 34111274 | population-based nationwide retrospective chart review, RDV 5 day course | Denmark, 1694 RDV plus DXM June through Dec 13 centers vs 1053 SOC only Feb through May 8 centers | 2. to 12.20 | late cohort RDV-DXM associated with reduced mortality and ventilation compared to early cohort SOC | the compared groups are not concurrent (SOC is early, RDV-DXM is late) |
| Ayodele | 34962924 | Retrospective cohort study comparing multiple treatments for inpatients, including RDV 5-10 days | US 26,192 pts 35% DXM, 15% RDV-DXM | 9.20 to 1.21 | no treatments appeared to alleviate the inpatient morbidity and mortality associated with COVID-19 |  |
| Chokkalingam |  | comparative effectiveness analysis using a matched cohort design in US-based hospital claims data for hospitalized patients with COVID-19 with or without evidence of treatment with RDV (5-10 days) during their hospital stay according to a pre-specified analytical plan | US hospital | 5.1.20 to 5.3.21 | compared 24,856 pts receiving RDV v 24,856 reference pts and found RDV associated with statistically significant reduction in D28 mortality in patients hospitalized with COVID-19 in each subgroup of baseline oxygen requirement |  |
| Elshaboury | 34589214 | retrospective chart review of inpatients who received RDV 5-10 days (no comparator) | a single hospital in Boston MA US | 5. to 10.20 | 164 RDV pts had outcomes comparable to those of clinical trials (7 and 12% mortality D14 and 28) | 5 d RDV; mortality rate decreased over analysis period possibly due to dexamethasone. |
| Garibaldi | 33760094 | retrospective comparative effectiveness research study, RDV 5-10 days | a 5-hospital system in Baltimore MD and Washington DC area | 3.4 to 8.29. 20 | 81% non-white cohort; 342 RDV recipients (184 corticosteroids, 158 RDV alone), RDV associated with faster recovery (5d v 7d) and steroid addition did not reduce hazard of 28d death |  |
| Gupte | 34983406 | retrospective analysis used data from an active surveillance program database of hospitalized patients with COVID-19 who were receiving RDV (5-10 days). | India | 7.9 to 10.15.20 | Of the 2329 patients included, 67.40% were men. Diabetes (29.69%) and hypertension (20.33%) were the most common co-morbidities. RDV is well tolerated and has an acceptable safety profile. The clinical outcome of cure/improvement was 84%, with a higher improvement in patients<60 years old and on standard low-flow oxygen. |  |

***** 3-day RDV is indicated and recommended by guidelines bodies such as NIH, to treat high-risk non-hospitalized patients to prevent disease progression. 5-day RDV is indicated and recommended to treat the hospitalized patients who had progressed to moderate or severe diseases. However, in this real-world analysis, the treatment duration was up to provider discretion.
