# Supplementary material for: Real-world evidence of survival benefit of remdesivir: study of 419 propensity score-matched patients hospitalized over the alpha and delta waves of COVID-19 in New Orleans, LA
Source: Front Med (Lausanne). 2024 May 16;11:1390164. doi: 10.3389/fmed.2024.1390164 (PMC11137210; doi:10.3389/fmed.2024.1390164)
Supplement: Supplementary file 2 [file Table_2.DOCX]

**Suppl. Table S2: FDA, WHO, IDSA guidelines in chronological order**

| Prelim results | Date of publication* | Date of accessed | Organism | Recommendation  in favor | Recommendation  against | Bases upon study | PMID |
| --- | --- | --- | --- | --- | --- | --- | --- |
|  | June 11^th^ 20 |  | FDA | Compassionate use |  | Grein et al.  N Engl J Med. | 32275812 |
| May 2020 | Oct. 22^nd^ 20 | Nov. 15 | FDA | EUA |  | ACTT-1 | 32445440 |
| Nov. 20^nd^ 2020 | Feb. 21^st^ 21 |  | WHO |  | WHO recommends against RDV use in COVID-19 patients | SOLIDARITY interim  N Engl J Med. | 33264556 |
|  | Oct. 22^nd^ 20 | Dec. 2^nd^ 20 | FDA | approval |  |  |  |
|  | April-June 20 |  | IDSA  v. 2.0 | # 8  Pro-use of RDV in severe COVID-19 (spO2< 94% on RA and require sup ox, MV or ECMO |  |  |  |
|  | Sept. 15 20 |  | IDSA  v. 3.2 | #10  RDV for less severe (oxygen suppl no MV) with a shorter 5 days course |  |  |  |
|  | Nov. 22 20 |  | IDSA  v.3.5 |  | # 11  against RDV if no supplement oxygen required |  |  |
|  | June 21st 21 |  | IDSA  v. 4.3 | # 10b  pro-RDV use for patients with ox sup requirements | #11  against the use of RDV in patients with MV/ECMO |  |  |
|  | Jan –Feb 22 |  | IDSA  v. 6.01 | #17  for the use of RDV in ambulatory |  |  |  |
|  | Jan –Feb 22 |  | IDSA  v. 7.01 | #13  for mild to moderate disease ambulatory or hospitalized |  |  |  |
| Apr. 22^nd^ 22 | Sep 22 |  | WHO | For the use of remdesivir in Severe or non-Severe COVID-19 patients who are at high risk of hospitalization |  | PINETREE  SOLIDARITY  Final  Lancet PMID | 35512728 |
