# Supplementary material for: Real-world evidence of survival benefit of remdesivir: study of 419 propensity score-matched patients hospitalized over the alpha and delta waves of COVID-19 in New Orleans, LA
Source: Front Med (Lausanne). 2024 May 16;11:1390164. doi: 10.3389/fmed.2024.1390164 (PMC11137210; doi:10.3389/fmed.2024.1390164)
Supplement: Supplementary file 3 [file Table_3.DOCX]

**Suppl. Table S3: Keywords used to identify medical conditions and medications.**

| **Condition/Medication** | **Dataset** | **Keyword** |
| --- | --- | --- |
| Congestive heart failure | DIAGNOSIS | ACUTE COMBINED SYSTOLIC (CONGESTIVE) AND DIASTOLIC (CONGESTIVE) HEART FAILURE  ACUTE DIASTOLIC (CONGESTIVE) HEART FAILURE  ACUTE ON CHRONIC COMBINED SYSTOLIC (CONGESTIVE) AND DIASTOLIC (CONGESTIVE) HEART FAILURE  ACUTE ON CHRONIC DIASTOLIC (CONGESTIVE) HEART FAILURE  ACUTE ON CHRONIC SYSTOLIC (CONGESTIVE) HEART FAILURE  ACUTE SYSTOLIC (CONGESTIVE) HEART FAILURE  CHRONIC COMBINED SYSTOLIC (CONGESTIVE) AND DIASTOLIC (CONGESTIVE) HEART FAILURE  CHRONIC DIASTOLIC (CONGESTIVE) HEART FAILURE  CHRONIC SYSTOLIC (CONGESTIVE) HEART FAILURE  UNSPECIFIED COMBINED SYSTOLIC (CONGESTIVE) AND DIASTOLIC (CONGESTIVE) HEART FAILURE  UNSPECIFIED DIASTOLIC (CONGESTIVE) HEART FAILURE  UNSPECIFIED SYSTOLIC (CONGESTIVE) HEART FAILURE |
|  | CONDITION | ACUTE ON CHRONIC COMBINED SYSTOLIC (CONGESTIVE) AND DIASTOLIC (CONGESTIVE) HEART FAILURE  ACUTE ON CHRONIC DIASTOLIC (CONGESTIVE) HEART FAILURE  ACUTE ON CHRONIC SYSTOLIC (CONGESTIVE) HEART FAILURE  ACUTE SYSTOLIC (CONGESTIVE) HEART FAILURE  CHRONIC COMBINED SYSTOLIC (CONGESTIVE) AND DIASTOLIC (CONGESTIVE) HEART FAILURE  CHRONIC DIASTOLIC (CONGESTIVE) HEART FAILURE  CHRONIC SYSTOLIC (CONGESTIVE) HEART FAILURE  UNSPECIFIED COMBINED SYSTOLIC (CONGESTIVE) AND DIASTOLIC (CONGESTIVE) HEART FAILURE  UNSPECIFIED DIASTOLIC (CONGESTIVE) HEART FAILURE  UNSPECIFIED SYSTOLIC (CONGESTIVE) HEART FAILURE |
| Myocardial infarction | DIAGNOSIS | ACUTE MYOCARDIAL INFARCTION, UNSPECIFIED  MYOCARDIAL INFARCTION TYPE 2  NON-ST ELEVATION (NSTEMI) MYOCARDIAL INFARCTION  OLD MYOCARDIAL INFARCTION  OTHER MYOCARDIAL INFARCTION TYPE  ST ELEVATION (STEMI) MYOCARDIAL INFARCTION INVOLVING LEFT ANTERIOR DESCENDING CORONARY ARTERY  ST ELEVATION (STEMI) MYOCARDIAL INFARCTION INVOLVING LEFT CIRCUMFLEX CORONARY ARTERY  ST ELEVATION (STEMI) MYOCARDIAL INFARCTION INVOLVING OTHER CORONARY ARTERY OF ANTERIOR WALL  ST ELEVATION (STEMI) MYOCARDIAL INFARCTION INVOLVING OTHER CORONARY ARTERY OF INFERIOR WALL  ST ELEVATION (STEMI) MYOCARDIAL INFARCTION INVOLVING OTHER SITES  ST ELEVATION (STEMI) MYOCARDIAL INFARCTION INVOLVING RIGHT CORONARY ARTERY  ST ELEVATION (STEMI) MYOCARDIAL INFARCTION OF UNSPECIFIED SITE  SUBSEQUENT NON-ST ELEVATION (NSTEMI) MYOCARDIAL INFARCTION |
|  | CONDITION | ACUTE MYOCARDIAL INFARCTION, UNSPECIFIED  MYOCARDIAL INFARCTION TYPE 2  NON-ST ELEVATION (NSTEMI) MYOCARDIAL INFARCTION  OLD MYOCARDIAL INFARCTION  ST ELEVATION (STEMI) MYOCARDIAL INFARCTION INVOLVING OTHER CORONARY ARTERY OF ANTERIOR WALL  ST ELEVATION (STEMI) MYOCARDIAL INFARCTION OF UNSPECIFIED SITE |
| Peripheral vascular disease | DIAGNOSIS | PERIPHERAL VASCULAR DISEASE, UNSPECIFIED |
|  | CONDITION | PERIPHERAL VASCULAR DISEASE, UNSPECIFIED |
| Cerebrovascular disease | DIAGNOSIS | ACUTE CEREBROVASCULAR INSUFFICIENCY  APHASIA FOLLOWING CEREBRAL INFARCTION  APHASIA FOLLOWING NONTRAUMATIC INTRACEREBRAL HEMORRHAGE  ATAXIA FOLLOWING CEREBRAL INFARCTION  ATAXIA FOLLOWING UNSPECIFIED CEREBROVASCULAR DISEASE  CEREBRAL ANEURYSM, NONRUPTURED  CEREBRAL ATHEROSCLEROSIS  CEREBRAL INFARCTION DUE TO EMBOLISM OF BASILAR ARTERY  CEREBRAL INFARCTION DUE TO EMBOLISM OF BILATERAL MIDDLE CEREBRAL ARTERIES  CEREBRAL INFARCTION DUE TO EMBOLISM OF BILATERAL POSTERIOR CEREBRAL ARTERIES  CEREBRAL INFARCTION DUE TO EMBOLISM OF LEFT CAROTID ARTERY  CEREBRAL INFARCTION DUE TO EMBOLISM OF LEFT MIDDLE CEREBRAL ARTERY  CEREBRAL INFARCTION DUE TO EMBOLISM OF OTHER CEREBRAL ARTERY  CEREBRAL INFARCTION DUE TO EMBOLISM OF RIGHT MIDDLE CEREBRAL ARTERY  CEREBRAL INFARCTION DUE TO EMBOLISM OF UNSPECIFIED CEREBRAL ARTERY  CEREBRAL INFARCTION DUE TO EMBOLISM OF UNSPECIFIED PRECEREBRAL ARTERY  CEREBRAL INFARCTION DUE TO THROMBOSIS OF OTHER CEREBRAL ARTERY  CEREBRAL INFARCTION DUE TO THROMBOSIS OF RIGHT VERTEBRAL ARTERY  CEREBRAL INFARCTION DUE TO THROMBOSIS OF UNSPECIFIED CEREBRAL ARTERY  CEREBRAL INFARCTION DUE TO UNSPECIFIED OCCLUSION OR STENOSIS OF LEFT ANTERIOR CEREBRAL ARTERY  CEREBRAL INFARCTION DUE TO UNSPECIFIED OCCLUSION OR STENOSIS OF LEFT CAROTID ARTERIES  CEREBRAL INFARCTION DUE TO UNSPECIFIED OCCLUSION OR STENOSIS OF LEFT MIDDLE CEREBRAL ARTERY  CEREBRAL INFARCTION DUE TO UNSPECIFIED OCCLUSION OR STENOSIS OF RIGHT CAROTID ARTERIES  CEREBRAL INFARCTION DUE TO UNSPECIFIED OCCLUSION OR STENOSIS OF RIGHT MIDDLE CEREBRAL ARTERY  CEREBRAL INFARCTION DUE TO UNSPECIFIED OCCLUSION OR STENOSIS OF UNSPECIFIED CEREBRAL ARTERY  CEREBRAL INFARCTION DUE TO UNSPECIFIED OCCLUSION OR STENOSIS OF UNSPECIFIED PRECEREBRAL ARTERIES  CEREBRAL INFARCTION, UNSPECIFIED  CEREBRAL ISCHEMIA  CEREBROVASCULAR DISEASE, UNSPECIFIED  DYSARTHRIA FOLLOWING CEREBRAL INFARCTION  DYSPHAGIA FOLLOWING CEREBRAL INFARCTION  DYSPHAGIA FOLLOWING NONTRAUMATIC INTRACEREBRAL HEMORRHAGE  DYSPHAGIA FOLLOWING UNSPECIFIED CEREBROVASCULAR DISEASE  FACIAL WEAKNESS FOLLOWING CEREBRAL INFARCTION  FACIAL WEAKNESS FOLLOWING NONTRAUMATIC INTRACEREBRAL HEMORRHAGE  CEREBROVASCULAR DISEASE AFFECTING UNSPECIFIED SIDE  HEMIPLEGIA AND HEMIPARESIS FOLLOWING CEREBRAL INFARCTION AFFECTING LEFT DOMINANT SIDE  HEMIPLEGIA AND HEMIPARESIS FOLLOWING CEREBRAL INFARCTION AFFECTING LEFT NON-DOMINANT SIDE  HEMIPLEGIA AND HEMIPARESIS FOLLOWING CEREBRAL INFARCTION AFFECTING RIGHT DOMINANT SIDE  HEMIPLEGIA AND HEMIPARESIS FOLLOWING CEREBRAL INFARCTION AFFECTING UNSPECIFIED SIDE  HEMIPLEGIA AND HEMIPARESIS FOLLOWING NONTRAUMATIC INTRACEREBRAL HEMORRHAGE AFFECTING LEFT NON-DOMINANT SIDE  HEMIPLEGIA AND HEMIPARESIS FOLLOWING NONTRAUMATIC INTRACEREBRAL HEMORRHAGE AFFECTING RIGHT DOMINANT SIDE  HEMIPLEGIA AND HEMIPARESIS FOLLOWING OTHER NONTRAUMATIC INTRACRANIAL HEMORRHAGE AFFECTING LEFT NON-DOMINANT SIDE  HEMIPLEGIA AND HEMIPARESIS FOLLOWING UNSPECIFIED CEREBROVASCULAR DISEASE AFFECTING LEFT NON-DOMINANT SIDE  HEMIPLEGIA AND HEMIPARESIS FOLLOWING UNSPECIFIED CEREBROVASCULAR DISEASE AFFECTING RIGHT DOMINANT SIDE  HEMIPLEGIA AND HEMIPARESIS FOLLOWING UNSPECIFIED CEREBROVASCULAR DISEASE AFFECTING UNSPECIFIED SIDE  HEMIPLEGIA, UNSPECIFIED AFFECTING LEFT NONDOMINANT SIDE  HEMIPLEGIA, UNSPECIFIED AFFECTING RIGHT DOMINANT SIDE  HEMIPLEGIA, UNSPECIFIED AFFECTING UNSPECIFIED SIDE  HYPERTENSIVE ENCEPHALOPATHY  MEMORY DEFICIT FOLLOWING CEREBRAL INFARCTION  MONOPLEGIA OF LOWER LIMB FOLLOWING CEREBRAL INFARCTION AFFECTING LEFT NON-DOMINANT SIDE  MONOPLEGIA OF LOWER LIMB FOLLOWING CEREBRAL INFARCTION AFFECTING RIGHT DOMINANT SIDE  MONOPLEGIA OF UPPER LIMB FOLLOWING CEREBRAL INFARCTION AFFECTING LEFT NON-DOMINANT SIDE  MONOPLEGIA OF UPPER LIMB FOLLOWING UNSPECIFIED CEREBROVASCULAR DISEASE AFFECTING RIGHT DOMINANT SIDE  MOYAMOYA DISEASE  NONTRAUMATIC CHRONIC SUBDURAL HEMORRHAGE  NONTRAUMATIC INTRACEREBRAL HEMORRHAGE IN HEMISPHERE, CORTICAL  NONTRAUMATIC INTRACEREBRAL HEMORRHAGE IN HEMISPHERE, SUBCORTICAL  NONTRAUMATIC INTRACEREBRAL HEMORRHAGE, INTRAVENTRICULAR  NONTRAUMATIC INTRACEREBRAL HEMORRHAGE, UNSPECIFIED  NONTRAUMATIC INTRACRANIAL HEMORRHAGE, UNSPECIFIED  NONTRAUMATIC SUBARACHNOID HEMORRHAGE FROM LEFT POSTERIOR COMMUNICATING ARTERY  NONTRAUMATIC SUBARACHNOID HEMORRHAGE, UNSPECIFIED  NONTRAUMATIC SUBDURAL HEMORRHAGE, UNSPECIFIED  OCCLUSION AND STENOSIS OF BASILAR ARTERY  OCCLUSION AND STENOSIS OF BILATERAL ANTERIOR CEREBRAL ARTERIES  OCCLUSION AND STENOSIS OF BILATERAL CAROTID ARTERIES  OCCLUSION AND STENOSIS OF LEFT CAROTID ARTERY  OCCLUSION AND STENOSIS OF LEFT MIDDLE CEREBRAL ARTERY  OCCLUSION AND STENOSIS OF LEFT VERTEBRAL ARTERY  OCCLUSION AND STENOSIS OF RIGHT CAROTID ARTERY  OCCLUSION AND STENOSIS OF RIGHT MIDDLE CEREBRAL ARTERY  OCCLUSION AND STENOSIS OF RIGHT POSTERIOR CEREBRAL ARTERY  OCCLUSION AND STENOSIS OF RIGHT VERTEBRAL ARTERY  OCCLUSION AND STENOSIS OF UNSPECIFIED CAROTID ARTERY  OCCLUSION AND STENOSIS OF UNSPECIFIED MIDDLE CEREBRAL ARTERY  OTHER CEREBRAL INFARCTION  OTHER CEREBRAL INFARCTION DUE TO OCCLUSION OR STENOSIS OF SMALL ARTERY  OTHER NONTRAUMATIC SUBARACHNOID HEMORRHAGE  OTHER SEQUELAE FOLLOWING UNSPECIFIED CEREBROVASCULAR DISEASE  OTHER SEQUELAE OF CEREBRAL INFARCTION  OTHER SEQUELAE OF NONTRAUMATIC INTRACEREBRAL HEMORRHAGE  OTHER SPEECH AND LANGUAGE DEFICITS FOLLOWING CEREBRAL INFARCTION  OTHER SYMPTOMS AND SIGNS INVOLVING COGNITIVE FUNCTIONS FOLLOWING CEREBRAL INFARCTION  POSTERIOR REVERSIBLE ENCEPHALOPATHY SYNDROME  UNSPECIFIED SEQUELAE OF CEREBRAL INFARCTION  UNSPECIFIED SEQUELAE OF UNSPECIFIED CEREBROVASCULAR DISEASE  UNSPECIFIED SYMPTOMS AND SIGNS INVOLVING COGNITIVE FUNCTIONS FOLLOWING CEREBRAL INFARCTION  UNSPECIFIED SYMPTOMS AND SIGNS INVOLVING COGNITIVE FUNCTIONS FOLLOWING UNSPECIFIED CEREBROVASCULAR DISEASE  VISUOSPATIAL DEFICIT AND SPATIAL NEGLECT FOLLOWING CEREBRAL INFARCTION |
|  | CONDITION | APHASIA FOLLOWING CEREBRAL INFARCTION  APHASIA FOLLOWING NONTRAUMATIC INTRACEREBRAL HEMORRHAGE  CEREBRAL AMYLOID ANGIOPATHY  CEREBRAL ANEURYSM, NONRUPTURED  CEREBRAL ATHEROSCLEROSIS  CEREBRAL INFARCTION DUE TO EMBOLISM OF BILATERAL MIDDLE CEREBRAL ARTERIES  CEREBRAL INFARCTION DUE TO EMBOLISM OF BILATERAL POSTERIOR CEREBRAL ARTERIES  CEREBRAL INFARCTION DUE TO EMBOLISM OF RIGHT CEREBELLAR ARTERY  CEREBRAL INFARCTION DUE TO EMBOLISM OF RIGHT MIDDLE CEREBRAL ARTERY  CEREBRAL INFARCTION DUE TO EMBOLISM OF UNSPECIFIED CEREBRAL ARTERY  CEREBRAL INFARCTION DUE TO THROMBOSIS OF RIGHT CEREBELLAR ARTERY  CEREBRAL INFARCTION DUE TO UNSPECIFIED OCCLUSION OR STENOSIS OF LEFT CAROTID ARTERIES  CEREBRAL INFARCTION DUE TO UNSPECIFIED OCCLUSION OR STENOSIS OF LEFT MIDDLE CEREBRAL ARTERY  CEREBRAL INFARCTION DUE TO UNSPECIFIED OCCLUSION OR STENOSIS OF RIGHT CAROTID ARTERIES  CEREBRAL INFARCTION DUE TO UNSPECIFIED OCCLUSION OR STENOSIS OF RIGHT MIDDLE CEREBRAL ARTERY  CEREBRAL INFARCTION, UNSPECIFIED  CEREBROVASCULAR DISEASE, UNSPECIFIED  DYSARTHRIA FOLLOWING CEREBRAL INFARCTION  DYSPHAGIA FOLLOWING CEREBRAL INFARCTION  DYSPHASIA FOLLOWING UNSPECIFIED CEREBROVASCULAR DISEASE  FACIAL WEAKNESS FOLLOWING CEREBRAL INFARCTION  FLACCID HEMIPLEGIA AFFECTING UNSPECIFIED SIDE  HEMIPLEGIA AND HEMIPARESIS FOLLOWING CEREBRAL INFARCTION AFFECTING LEFT NON-DOMINANT SIDE  HEMIPLEGIA AND HEMIPARESIS FOLLOWING CEREBRAL INFARCTION AFFECTING RIGHT DOMINANT SIDE  HEMIPLEGIA, UNSPECIFIED AFFECTING LEFT NONDOMINANT SIDE  HEMIPLEGIA, UNSPECIFIED AFFECTING RIGHT DOMINANT SIDE  HEMIPLEGIA, UNSPECIFIED AFFECTING UNSPECIFIED SIDE  MOYAMOYA DISEASE  NONTRAUMATIC CHRONIC SUBDURAL HEMORRHAGE  NONTRAUMATIC INTRACEREBRAL HEMORRHAGE, INTRAVENTRICULAR  NONTRAUMATIC INTRACEREBRAL HEMORRHAGE, UNSPECIFIED  NONTRAUMATIC INTRACRANIAL HEMORRHAGE, UNSPECIFIED  NONTRAUMATIC SUBARACHNOID HEMORRHAGE, UNSPECIFIED  NONTRAUMATIC SUBDURAL HEMORRHAGE, UNSPECIFIED  OCCLUSION AND STENOSIS OF BILATERAL CAROTID ARTERIES  OCCLUSION AND STENOSIS OF LEFT CAROTID ARTERY  OCCLUSION AND STENOSIS OF LEFT MIDDLE CEREBRAL ARTERY  OCCLUSION AND STENOSIS OF RIGHT CAROTID ARTERY  OCCLUSION AND STENOSIS OF UNSPECIFIED CAROTID ARTERY  OTHER SEQUELAE FOLLOWING UNSPECIFIED CEREBROVASCULAR DISEASE  OTHER SEQUELAE OF CEREBRAL INFARCTION  UNSPECIFIED SEQUELAE OF CEREBRAL INFARCTION |
| Chronic pulmonary disease | DIAGNOSIS | ADVERSE EFFECT OF ANTIASTHMATICS, INITIAL ENCOUNTER  BRONCHIECTASIS WITH (ACUTE) EXACERBATION  BRONCHIECTASIS WITH ACUTE LOWER RESPIRATORY INFECTION  BRONCHIECTASIS, UNCOMPLICATED  CENTRILOBULAR EMPHYSEMA  CHRONIC OBSTRUCTIVE PULMONARY DISEASE WITH (ACUTE) EXACERBATION  CHRONIC OBSTRUCTIVE PULMONARY DISEASE WITH (ACUTE) LOWER RESPIRATORY INFECTION  CHRONIC OBSTRUCTIVE PULMONARY DISEASE, UNSPECIFIED  CHRONIC PULMONARY EMBOLISM  CHRONIC THROMBOEMBOLIC PULMONARY HYPERTENSION  CYSTIC FIBROSIS CARRIER  EMPHYSEMA (SUBCUTANEOUS) RESULTING FROM A PROCEDURE, INITIAL ENCOUNTER  EMPHYSEMA, UNSPECIFIED  FAMILY HISTORY OF ASTHMA AND OTHER CHRONIC LOWER RESPIRATORY DISEASES  IDIOPATHIC PULMONARY FIBROSIS  INTERSTITIAL EMPHYSEMA  INTERSTITIAL PULMONARY DISEASE, UNSPECIFIED  MILD INTERMITTENT ASTHMA WITH (ACUTE) EXACERBATION  MILD INTERMITTENT ASTHMA, UNCOMPLICATED  MILD PERSISTENT ASTHMA, UNCOMPLICATED  MODERATE PERSISTENT ASTHMA WITH (ACUTE) EXACERBATION  MODERATE PERSISTENT ASTHMA, UNCOMPLICATED  OTHER ASTHMA  OTHER EMPHYSEMA  OTHER PULMONARY EMBOLISM WITH ACUTE COR PULMONALE  OTHER PULMONARY EMBOLISM WITHOUT ACUTE COR PULMONALE  OTHER SECONDARY PULMONARY HYPERTENSION  OTHER SPECIFIED INTERSTITIAL PULMONARY DISEASES  PERSONAL HISTORY OF PULMONARY EMBOLISM  PRIMARY PULMONARY HYPERTENSION  PULMONARY HYPERTENSION DUE TO LEFT HEART DISEASE  PULMONARY HYPERTENSION DUE TO LUNG DISEASES AND HYPOXIA  PULMONARY HYPERTENSION, UNSPECIFIED  SEPTIC PULMONARY EMBOLISM WITHOUT ACUTE COR PULMONALE  SEVERE PERSISTENT ASTHMA WITH (ACUTE) EXACERBATION  SEVERE PERSISTENT ASTHMA, UNCOMPLICATED  SIMPLE CHRONIC BRONCHITIS  SINGLE SUBSEGMENTAL PULMONARY EMBOLISM WITHOUT ACUTE COR PULMONALE  TRAUMATIC SUBCUTANEOUS EMPHYSEMA, INITIAL ENCOUNTER  UNSPECIFIED ASTHMA WITH (ACUTE) EXACERBATION  UNSPECIFIED ASTHMA WITH STATUS ASTHMATICUS  UNSPECIFIED ASTHMA, UNCOMPLICATED  UNSPECIFIED CHRONIC BRONCHITIS |
|  | CONDITION | ADVERSE EFFECT OF ANTIASTHMATICS, INITIAL ENCOUNTER  CHRONIC OBSTRUCTIVE PULMONARY DISEASE WITH (ACUTE) EXACERBATION  CHRONIC OBSTRUCTIVE PULMONARY DISEASE WITH (ACUTE) LOWER RESPIRATORY INFECTION  CHRONIC OBSTRUCTIVE PULMONARY DISEASE, UNSPECIFIED  CHRONIC PULMONARY EMBOLISM  EMPHYSEMA, UNSPECIFIED  IDIOPATHIC PULMONARY FIBROSIS  INTERSTITIAL EMPHYSEMA  INTERSTITIAL PULMONARY DISEASE, UNSPECIFIED  MILD INTERMITTENT ASTHMA WITH (ACUTE) EXACERBATION  MILD INTERMITTENT ASTHMA, UNCOMPLICATED  OTHER PULMONARY EMBOLISM WITHOUT ACUTE COR PULMONALE  PERSONAL HISTORY OF PULMONARY EMBOLISM  PULMONARY HYPERTENSION DUE TO LEFT HEART DISEASE  PULMONARY HYPERTENSION, UNSPECIFIED  SEVERE PERSISTENT ASTHMA WITH (ACUTE) EXACERBATION  SINGLE SUBSEGMENTAL PULMONARY EMBOLISM WITHOUT ACUTE COR PULMONALE  UNSPECIFIED ASTHMA WITH (ACUTE) EXACERBATION  UNSPECIFIED ASTHMA, UNCOMPLICATED  UNSPECIFIED CHRONIC BRONCHITIS |
| Diabetes | DIAGNOSIS | OTHER SPECIFIED DIABETES MELLITUS WITH PROLIFERATIVE DIABETIC RETINOPATHY WITHOUT MACULAR EDEMA, RIGHT EYE  OTHER SPECIFIED DIABETES MELLITUS WITH SEVERE NONPROLIFERATIVE DIABETIC RETINOPATHY WITHOUT MACULAR EDEMA, LEFT EYE  OTHER SPECIFIED DIABETES MELLITUS WITH UNSPECIFIED DIABETIC RETINOPATHY WITHOUT MACULAR EDEMA  OTHER SPECIFIED DIABETES MELLITUS WITHOUT COMPLICATIONS TYPE 1 DIABETES MELLITUS WITH DIABETIC AUTONOMIC (POLY)NEUROPATHY  TYPE 1 DIABETES MELLITUS WITH DIABETIC CATARACT  TYPE 1 DIABETES MELLITUS WITH DIABETIC CHRONIC KIDNEY DISEASE  TYPE 1 DIABETES MELLITUS WITH DIABETIC NEPHROPATHY  TYPE 1 DIABETES MELLITUS WITH DIABETIC POLYNEUROPATHY  TYPE 1 DIABETES MELLITUS WITH HYPERGLYCEMIA  TYPE 1 DIABETES MELLITUS WITH HYPOGLYCEMIA WITHOUT COMA  TYPE 1 DIABETES MELLITUS WITH KETOACIDOSIS WITH COMA  TYPE 1 DIABETES MELLITUS WITH KETOACIDOSIS WITHOUT COMA  TYPE 1 DIABETES MELLITUS WITH MILD NONPROLIFERATIVE DIABETIC RETINOPATHY WITHOUT MACULAR EDEMA, BILATERAL  TYPE 1 DIABETES MELLITUS WITH MODERATE NONPROLIFERATIVE DIABETIC RETINOPATHY WITHOUT MACULAR EDEMA, UNSPECIFIED EYE  TYPE 1 DIABETES MELLITUS WITH OTHER DIABETIC KIDNEY COMPLICATION  TYPE 1 DIABETES MELLITUS WITH OTHER SPECIFIED COMPLICATION  TYPE 1 DIABETES MELLITUS WITH PROLIFERATIVE DIABETIC RETINOPATHY WITH TRACTION RETINAL DETACHMENT NOT INVOLVING THE MACULA, LEFT EYE  TYPE 1 DIABETES MELLITUS WITH PROLIFERATIVE DIABETIC RETINOPATHY WITHOUT MACULAR EDEMA, BILATERAL  TYPE 1 DIABETES MELLITUS WITH SEVERE NONPROLIFERATIVE DIABETIC RETINOPATHY WITH MACULAR EDEMA, BILATERAL  TYPE 1 DIABETES MELLITUS WITH UNSPECIFIED DIABETIC RETINOPATHY WITHOUT MACULAR EDEMA  TYPE 1 DIABETES MELLITUS WITHOUT COMPLICATIONS  TYPE 2 DIABETES MELLITUS WITH DIABETIC AUTONOMIC (POLY)NEUROPATHY  TYPE 2 DIABETES MELLITUS WITH DIABETIC CATARACT  TYPE 2 DIABETES MELLITUS WITH DIABETIC CHRONIC KIDNEY DISEASE  TYPE 2 DIABETES MELLITUS WITH DIABETIC DERMATITIS  TYPE 2 DIABETES MELLITUS WITH DIABETIC MONONEUROPATHY  TYPE 2 DIABETES MELLITUS WITH DIABETIC NEPHROPATHY  TYPE 2 DIABETES MELLITUS WITH DIABETIC NEUROPATHIC ARTHROPATHY  TYPE 2 DIABETES MELLITUS WITH DIABETIC NEUROPATHY, UNSPECIFIED  TYPE 2 DIABETES MELLITUS WITH DIABETIC PERIPHERAL ANGIOPATHY WITH GANGRENE  TYPE 2 DIABETES MELLITUS WITH DIABETIC PERIPHERAL ANGIOPATHY WITHOUT GANGRENE  TYPE 2 DIABETES MELLITUS WITH DIABETIC POLYNEUROPATHY  TYPE 2 DIABETES MELLITUS WITH FOOT ULCER  TYPE 2 DIABETES MELLITUS WITH HYPERGLYCEMIA  TYPE 2 DIABETES MELLITUS WITH HYPEROSMOLARITY WITHOUT NONKETOTIC HYPERGLYCEMIC-HYPEROSMOLAR COMA (NKHHC)  TYPE 2 DIABETES MELLITUS WITH HYPOGLYCEMIA WITH COMA  TYPE 2 DIABETES MELLITUS WITH HYPOGLYCEMIA WITHOUT COMA  TYPE 2 DIABETES MELLITUS WITH KETOACIDOSIS WITH COMA  TYPE 2 DIABETES MELLITUS WITH KETOACIDOSIS WITHOUT COMA  TYPE 2 DIABETES MELLITUS WITH MILD NONPROLIFERATIVE DIABETIC RETINOPATHY WITH MACULAR EDEMA, BILATERAL  TYPE 2 DIABETES MELLITUS WITH MILD NONPROLIFERATIVE DIABETIC RETINOPATHY WITH MACULAR EDEMA, RIGHT EYE  TYPE 2 DIABETES MELLITUS WITH MILD NONPROLIFERATIVE DIABETIC RETINOPATHY WITH MACULAR EDEMA, UNSPECIFIED EYE  TYPE 2 DIABETES MELLITUS WITH MILD NONPROLIFERATIVE DIABETIC RETINOPATHY WITHOUT MACULAR EDEMA, BILATERAL  TYPE 2 DIABETES MELLITUS WITH MILD NONPROLIFERATIVE DIABETIC RETINOPATHY WITHOUT MACULAR EDEMA, LEFT EYE  TYPE 2 DIABETES MELLITUS WITH MILD NONPROLIFERATIVE DIABETIC RETINOPATHY WITHOUT MACULAR EDEMA, RIGHT EYE  TYPE 2 DIABETES MELLITUS WITH MILD NONPROLIFERATIVE DIABETIC RETINOPATHY WITHOUT MACULAR EDEMA, UNSPECIFIED EYE  TYPE 2 DIABETES MELLITUS WITH MODERATE NONPROLIFERATIVE DIABETIC RETINOPATHY WITH MACULAR EDEMA, BILATERAL  TYPE 2 DIABETES MELLITUS WITH MODERATE NONPROLIFERATIVE DIABETIC RETINOPATHY WITH MACULAR EDEMA, LEFT EYE  TYPE 2 DIABETES MELLITUS WITH MODERATE NONPROLIFERATIVE DIABETIC RETINOPATHY WITH MACULAR EDEMA, RIGHT EYE  TYPE 2 DIABETES MELLITUS WITH MODERATE NONPROLIFERATIVE DIABETIC RETINOPATHY WITHOUT MACULAR EDEMA, BILATERAL  TYPE 2 DIABETES MELLITUS WITH MODERATE NONPROLIFERATIVE DIABETIC RETINOPATHY WITHOUT MACULAR EDEMA, LEFT EYE  TYPE 2 DIABETES MELLITUS WITH MODERATE NONPROLIFERATIVE DIABETIC RETINOPATHY WITHOUT MACULAR EDEMA, RIGHT EYE  TYPE 2 DIABETES MELLITUS WITH MODERATE NONPROLIFERATIVE DIABETIC RETINOPATHY WITHOUT MACULAR EDEMA, UNSPECIFIED EYE  TYPE 2 DIABETES MELLITUS WITH OTHER CIRCULATORY COMPLICATIONS  TYPE 2 DIABETES MELLITUS WITH OTHER DIABETIC KIDNEY COMPLICATION  TYPE 2 DIABETES MELLITUS WITH OTHER DIABETIC NEUROLOGICAL COMPLICATION  TYPE 2 DIABETES MELLITUS WITH OTHER DIABETIC OPHTHALMIC COMPLICATION  TYPE 2 DIABETES MELLITUS WITH OTHER SKIN COMPLICATIONS  TYPE 2 DIABETES MELLITUS WITH OTHER SKIN ULCER  TYPE 2 DIABETES MELLITUS WITH OTHER SPECIFIED COMPLICATION  TYPE 2 DIABETES MELLITUS WITH PROLIFERATIVE DIABETIC RETINOPATHY WITH MACULAR EDEMA, BILATERAL  TYPE 2 DIABETES MELLITUS WITH PROLIFERATIVE DIABETIC RETINOPATHY WITH MACULAR EDEMA, LEFT EYE  TYPE 2 DIABETES MELLITUS WITH PROLIFERATIVE DIABETIC RETINOPATHY WITH MACULAR EDEMA, RIGHT EYE  TYPE 2 DIABETES MELLITUS WITH PROLIFERATIVE DIABETIC RETINOPATHY WITH MACULAR EDEMA, UNSPECIFIED EYE  TYPE 2 DIABETES MELLITUS WITH PROLIFERATIVE DIABETIC RETINOPATHY WITH TRACTION RETINAL DETACHMENT NOT INVOLVING THE MACULA, LEFT EYE  TYPE 2 DIABETES MELLITUS WITH PROLIFERATIVE DIABETIC RETINOPATHY WITHOUT MACULAR EDEMA, BILATERAL  TYPE 2 DIABETES MELLITUS WITH PROLIFERATIVE DIABETIC RETINOPATHY WITHOUT MACULAR EDEMA, LEFT EYE  TYPE 2 DIABETES MELLITUS WITH PROLIFERATIVE DIABETIC RETINOPATHY WITHOUT MACULAR EDEMA, RIGHT EYE  TYPE 2 DIABETES MELLITUS WITH PROLIFERATIVE DIABETIC RETINOPATHY WITHOUT MACULAR EDEMA, UNSPECIFIED EYE  TYPE 2 DIABETES MELLITUS WITH SEVERE NONPROLIFERATIVE DIABETIC RETINOPATHY WITH MACULAR EDEMA, BILATERAL  TYPE 2 DIABETES MELLITUS WITH SEVERE NONPROLIFERATIVE DIABETIC RETINOPATHY WITHOUT MACULAR EDEMA, BILATERAL  TYPE 2 DIABETES MELLITUS WITH SEVERE NONPROLIFERATIVE DIABETIC RETINOPATHY WITHOUT MACULAR EDEMA, RIGHT EYE  TYPE 2 DIABETES MELLITUS WITH STABLE PROLIFERATIVE DIABETIC RETINOPATHY, UNSPECIFIED EYE  TYPE 2 DIABETES MELLITUS WITH UNSPECIFIED COMPLICATIONS  TYPE 2 DIABETES MELLITUS WITH UNSPECIFIED DIABETIC RETINOPATHY WITH MACULAR EDEMA  TYPE 2 DIABETES MELLITUS WITH UNSPECIFIED DIABETIC RETINOPATHY WITHOUT MACULAR EDEMA  TYPE 2 DIABETES MELLITUS WITHOUT COMPLICATIONS |
|  | CONDITION | TYPE 1 DIABETES MELLITUS WITH DIABETIC CHRONIC KIDNEY DISEASE  TYPE 1 DIABETES MELLITUS WITH DIABETIC NEPHROPATHY  TYPE 1 DIABETES MELLITUS WITH FOOT ULCER  TYPE 1 DIABETES MELLITUS WITH HYPERGLYCEMIA  TYPE 1 DIABETES MELLITUS WITH HYPOGLYCEMIA WITHOUT COMA  TYPE 1 DIABETES MELLITUS WITH KETOACIDOSIS WITH COMA  TYPE 1 DIABETES MELLITUS WITH KETOACIDOSIS WITHOUT COMA  TYPE 1 DIABETES MELLITUS WITHOUT COMPLICATIONS  TYPE 2 DIABETES MELLITUS WITH DIABETIC AUTONOMIC (POLY)NEUROPATHY  TYPE 2 DIABETES MELLITUS WITH DIABETIC CATARACT  TYPE 2 DIABETES MELLITUS WITH DIABETIC CHRONIC KIDNEY DISEASE  TYPE 2 DIABETES MELLITUS WITH DIABETIC NEPHROPATHY  TYPE 2 DIABETES MELLITUS WITH DIABETIC NEUROPATHIC ARTHROPATHY  TYPE 2 DIABETES MELLITUS WITH DIABETIC NEUROPATHY, UNSPECIFIED  TYPE 2 DIABETES MELLITUS WITH DIABETIC PERIPHERAL ANGIOPATHY WITH GANGRENE  TYPE 2 DIABETES MELLITUS WITH DIABETIC PERIPHERAL ANGIOPATHY WITHOUT GANGRENE  TYPE 2 DIABETES MELLITUS WITH DIABETIC POLYNEUROPATHY  TYPE 2 DIABETES MELLITUS WITH FOOT ULCER  TYPE 2 DIABETES MELLITUS WITH HYPERGLYCEMIA  TYPE 2 DIABETES MELLITUS WITH HYPEROSMOLARITY WITHOUT NONKETOTIC HYPERGLYCEMIC-HYPEROSMOLAR COMA (NKHHC)  TYPE 2 DIABETES MELLITUS WITH HYPOGLYCEMIA WITHOUT COMA  TYPE 2 DIABETES MELLITUS WITH KETOACIDOSIS WITHOUT COMA  TYPE 2 DIABETES MELLITUS WITH MILD NONPROLIFERATIVE DIABETIC RETINOPATHY WITHOUT MACULAR EDEMA, BILATERAL  TYPE 2 DIABETES MELLITUS WITH MILD NONPROLIFERATIVE DIABETIC RETINOPATHY WITHOUT MACULAR EDEMA, UNSPECIFIED EYE  TYPE 2 DIABETES MELLITUS WITH OTHER SPECIFIED COMPLICATION  TYPE 2 DIABETES MELLITUS WITH UNSPECIFIED COMPLICATIONS  TYPE 2 DIABETES MELLITUS WITH UNSPECIFIED DIABETIC RETINOPATHY WITHOUT MACULAR EDEMA  TYPE 2 DIABETES MELLITUS WITHOUT COMPLICATIONS |
| Renal disease | DIAGNOSIS | CHRONIC KIDNEY DISEASE, STAGE 1  CHRONIC KIDNEY DISEASE, STAGE 2 (MILD)  CHRONIC KIDNEY DISEASE, STAGE 3 (MODERATE)  CHRONIC KIDNEY DISEASE, STAGE 3 UNSPECIFIED  CHRONIC KIDNEY DISEASE, STAGE 3A  CHRONIC KIDNEY DISEASE, STAGE 3B  CHRONIC KIDNEY DISEASE, STAGE 4 (SEVERE) |
|  | CONDITION | CHRONIC KIDNEY DISEASE, STAGE 2 (MILD)  CHRONIC KIDNEY DISEASE, STAGE 3 (MODERATE)  CHRONIC KIDNEY DISEASE, STAGE 3 UNSPECIFIED  CHRONIC KIDNEY DISEASE, STAGE 3A  CHRONIC KIDNEY DISEASE, STAGE 3B  CHRONIC KIDNEY DISEASE, STAGE 4 (SEVERE) |
| Liver disease | DIAGNOSIS | ACUTE AND SUBACUTE HEPATIC FAILURE WITHOUT COMA  ACUTE HEPATITIS B WITHOUT DELTA-AGENT AND WITHOUT HEPATIC COMA  ACUTE HEPATITIS C WITHOUT HEPATIC COMA  ACUTE VIRAL HEPATITIS, UNSPECIFIED  ALCOHOLIC CIRRHOSIS OF LIVER WITH ASCITES  ALCOHOLIC CIRRHOSIS OF LIVER WITHOUT ASCITES  ALCOHOLIC HEPATIC FAILURE WITHOUT COMA  ALCOHOLIC HEPATITIS WITH ASCITES  ALCOHOLIC HEPATITIS WITHOUT ASCITES  ALCOHOLIC LIVER DISEASE, UNSPECIFIED  AUTOIMMUNE HEPATITIS  CHRONIC HEPATIC FAILURE WITHOUT COMA  CHRONIC PASSIVE CONGESTION OF LIVER  CHRONIC VIRAL HEPATITIS B WITHOUT DELTA-AGENT  CHRONIC VIRAL HEPATITIS C  FATTY (CHANGE OF) LIVER, NOT ELSEWHERE CLASSIFIED  LIVER CELL CARCINOMA  LIVER DISEASE, UNSPECIFIED  MALIGNANT NEOPLASM OF HEPATIC FLEXURE  NONALCOHOLIC STEATOHEPATITIS (NASH)  OTHER CIRRHOSIS OF LIVER  SECONDARY MALIGNANT NEOPLASM OF LIVER AND INTRAHEPATIC BILE DUCT  UNSPECIFIED CIRRHOSIS OF LIVER |
|  | CONDITION | ACUTE AND SUBACUTE HEPATIC FAILURE WITHOUT COMA  ACUTE VIRAL HEPATITIS, UNSPECIFIED  ALCOHOLIC CIRRHOSIS OF LIVER WITH ASCITES  ALCOHOLIC CIRRHOSIS OF LIVER WITHOUT ASCITES  ALCOHOLIC HEPATIC FAILURE WITHOUT COMA  ALCOHOLIC HEPATITIS WITHOUT ASCITES  AUTOIMMUNE HEPATITIS  CHRONIC PASSIVE CONGESTION OF LIVER  CHRONIC VIRAL HEPATITIS B WITHOUT DELTA-AGENT  CHRONIC VIRAL HEPATITIS C  FATTY (CHANGE OF) LIVER, NOT ELSEWHERE CLASSIFIED  LIVER CELL CARCINOMA  LIVER DISEASE, UNSPECIFIED  NONALCOHOLIC STEATOHEPATITIS (NASH)  SECONDARY MALIGNANT NEOPLASM OF LIVER AND INTRAHEPATIC BILE DUCT  UNSPECIFIED CIRRHOSIS OF LIVER |
| Peptic ulcer disease | DIAGNOSIS | ACUTE GASTRIC ULCER WITH HEMORRHAGE  ACUTE GASTRIC ULCER WITH PERFORATION  CHRONIC GASTRIC ULCER WITHOUT HEMORRHAGE OR PERFORATION  CHRONIC OR UNSPECIFIED DUODENAL ULCER WITH HEMORRHAGE  CHRONIC OR UNSPECIFIED DUODENAL ULCER WITH PERFORATION  CHRONIC OR UNSPECIFIED GASTRIC ULCER WITH HEMORRHAGE  CHRONIC OR UNSPECIFIED GASTRIC ULCER WITH PERFORATION  CHRONIC OR UNSPECIFIED PEPTIC ULCER, SITE UNSPECIFIED, WITH HEMORRHAGE  DUODENAL ULCER, UNSPECIFIED AS ACUTE OR CHRONIC, WITHOUT HEMORRHAGE OR PERFORATION  GASTRIC ULCER, UNSPECIFIED AS ACUTE OR CHRONIC, WITHOUT HEMORRHAGE OR PERFORATION  GASTROJEJUNAL ULCER, UNSPECIFIED AS ACUTE OR CHRONIC, WITHOUT HEMORRHAGE OR PERFORATION  PEPTIC ULCER, SITE UNSPECIFIED, UNSPECIFIED AS ACUTE OR CHRONIC, WITHOUT HEMORRHAGE OR PERFORATION  PERSONAL HISTORY OF PEPTIC ULCER DISEASE |
|  | CONDITION | CHRONIC OR UNSPECIFIED PEPTIC ULCER, SITE UNSPECIFIED, WITH HEMORRHAGE  GASTRIC ULCER, UNSPECIFIED AS ACUTE OR CHRONIC, WITHOUT HEMORRHAGE OR PERFORATION  PEPTIC ULCER, SITE UNSPECIFIED, UNSPECIFIED AS ACUTE OR CHRONIC, WITHOUT HEMORRHAGE OR PERFORATION  PERSONAL HISTORY OF PEPTIC ULCER DISEASE |
| HIV/AIDS | DIAGNOSIS | HUMAN IMMUNODEFICIENCY VIRUS [HIV] DISEASE |
|  | CONDITION | HUMAN IMMUNODEFICIENCY VIRUS [HIV] DISEASE |
| Lymphoma | DIAGNOSIS | DIFFUSE LARGE B-CELL LYMPHOMA, EXTRANODAL AND SOLID ORGAN SITES  DIFFUSE LARGE B-CELL LYMPHOMA, UNSPECIFIED SITE  FOLLICULAR LYMPHOMA GRADE I, UNSPECIFIED SITE  FOLLICULAR LYMPHOMA GRADE III, UNSPECIFIED, UNSPECIFIED SITE  FOLLICULAR LYMPHOMA GRADE IIIA, LYMPH NODES OF HEAD, FACE, AND NECK  FOLLICULAR LYMPHOMA GRADE IIIA, UNSPECIFIED SITE  FOLLICULAR LYMPHOMA GRADE IIIB, LYMPH NODES OF MULTIPLE SITES  FOLLICULAR LYMPHOMA, UNSPECIFIED, UNSPECIFIED SITE  HODGKIN LYMPHOMA, UNSPECIFIED, UNSPECIFIED SITE  LYMPHOBLASTIC (DIFFUSE) LYMPHOMA, UNSPECIFIED SITE  MANTLE CELL LYMPHOMA, INTRA-ABDOMINAL LYMPH NODES  MANTLE CELL LYMPHOMA, LYMPH NODES OF MULTIPLE SITES  MANTLE CELL LYMPHOMA, UNSPECIFIED SITE  NODULAR SCLEROSIS HODGKIN LYMPHOMA, EXTRANODAL AND SOLID ORGAN SITES  NODULAR SCLEROSIS HODGKIN LYMPHOMA, INTRATHORACIC LYMPH NODES  NODULAR SCLEROSIS HODGKIN LYMPHOMA, UNSPECIFIED SITE  NON-HODGKIN LYMPHOMA, UNSPECIFIED, UNSPECIFIED SITE  OTHER HODGKIN LYMPHOMA, UNSPECIFIED SITE  OTHER SPECIFIED TYPES OF NON-HODGKIN LYMPHOMA, EXTRANODAL AND SOLID ORGAN SITES  OTHER SPECIFIED TYPES OF NON-HODGKIN LYMPHOMA, LYMPH NODES OF HEAD, FACE, AND NECK  OTHER SPECIFIED TYPES OF NON-HODGKIN LYMPHOMA, UNSPECIFIED SITE  PERSONAL HISTORY OF HODGKIN LYMPHOMA  PERSONAL HISTORY OF NON-HODGKIN LYMPHOMAS  UNSPECIFIED B-CELL LYMPHOMA, UNSPECIFIED SITE |
|  | CONDITION | DIFFUSE LARGE B-CELL LYMPHOMA, EXTRANODAL AND SOLID ORGAN SITES  FOLLICULAR LYMPHOMA, UNSPECIFIED, UNSPECIFIED SITE  HODGKIN LYMPHOMA, UNSPECIFIED, UNSPECIFIED SITE  NODULAR SCLEROSIS HODGKIN LYMPHOMA, UNSPECIFIED SITE  NON-HODGKIN LYMPHOMA, UNSPECIFIED, UNSPECIFIED SITE  OTHER SPECIFIED TYPES OF NON-HODGKIN LYMPHOMA, LYMPH NODES OF HEAD, FACE, AND NECK  PERSONAL HISTORY OF NON-HODGKIN LYMPHOMAS |
| Leukemia | DIAGNOSIS | ACUTE LEUKEMIA OF UNSPECIFIED CELL TYPE NOT HAVING ACHIEVED REMISSION  ACUTE LYMPHOBLASTIC LEUKEMIA NOT HAVING ACHIEVED REMISSION  ACUTE LYMPHOBLASTIC LEUKEMIA, IN RELAPSE  ACUTE LYMPHOBLASTIC LEUKEMIA, IN REMISSION  ACUTE MYELOBLASTIC LEUKEMIA, IN REMISSION  ACUTE MYELOBLASTIC LEUKEMIA, NOT HAVING ACHIEVED REMISSION  ACUTE PROMYELOCYTIC LEUKEMIA, NOT HAVING ACHIEVED REMISSION  CHRONIC LYMPHOCYTIC LEUKEMIA OF B-CELL TYPE NOT HAVING ACHIEVED REMISSION  CHRONIC MYELOID LEUKEMIA, BCR/ABL-POSITIVE, IN REMISSION  CHRONIC MYELOID LEUKEMIA, BCR/ABL-POSITIVE, NOT HAVING ACHIEVED REMISSION  CHRONIC MYELOMONOCYTIC LEUKEMIA NOT HAVING ACHIEVED REMISSION  FAMILY HISTORY OF LEUKEMIA  LEUKEMIA, UNSPECIFIED NOT HAVING ACHIEVED REMISSION  MYELOID LEUKEMIA, UNSPECIFIED, NOT HAVING ACHIEVED REMISSION  PERSONAL HISTORY OF LEUKEMIA  PROLYMPHOCYTIC LEUKEMIA OF T-CELL TYPE NOT HAVING ACHIEVED REMISSION |
|  | CONDITION | ACUTE LYMPHOBLASTIC LEUKEMIA NOT HAVING ACHIEVED REMISSION  ACUTE MYELOBLASTIC LEUKEMIA, NOT HAVING ACHIEVED REMISSION  ACUTE PROMYELOCYTIC LEUKEMIA, NOT HAVING ACHIEVED REMISSION  CHRONIC LYMPHOCYTIC LEUKEMIA OF B-CELL TYPE NOT HAVING ACHIEVED REMISSION  CHRONIC MYELOID LEUKEMIA, BCR/ABL-POSITIVE, IN REMISSION  CHRONIC MYELOID LEUKEMIA, BCR/ABL-POSITIVE, NOT HAVING ACHIEVED REMISSION  LEUKEMIA, UNSPECIFIED NOT HAVING ACHIEVED REMISSION  PERSONAL HISTORY OF LEUKEMIA  PROLYMPHOCYTIC LEUKEMIA OF T-CELL TYPE NOT HAVING ACHIEVED REMISSION |
| Solid tumor | DIAGNOSIS | ACUTE LEUKEMIA OF UNSPECIFIED CELL TYPE NOT HAVING ACHIEVED REMISSION  ACUTE LYMPHOBLASTIC LEUKEMIA NOT HAVING ACHIEVED REMISSION  ACUTE LYMPHOBLASTIC LEUKEMIA, IN RELAPSE  ACUTE LYMPHOBLASTIC LEUKEMIA, IN REMISSION  ACUTE MYELOBLASTIC LEUKEMIA, IN REMISSION  ACUTE MYELOBLASTIC LEUKEMIA, NOT HAVING ACHIEVED REMISSION  ACUTE PROMYELOCYTIC LEUKEMIA, NOT HAVING ACHIEVED REMISSION  AGRANULOCYTOSIS SECONDARY TO CANCER CHEMOTHERAPY  BASAL CELL CARCINOMA OF SKIN OF NOSE  BASAL CELL CARCINOMA OF SKIN OF SCALP AND NECK  CHRONIC LYMPHOCYTIC LEUKEMIA OF B-CELL TYPE NOT HAVING ACHIEVED REMISSION  CHRONIC MYELOID LEUKEMIA, BCR/ABL-POSITIVE, IN REMISSION  CHRONIC MYELOID LEUKEMIA, BCR/ABL-POSITIVE, NOT HAVING ACHIEVED REMISSION  CHRONIC MYELOMONOCYTIC LEUKEMIA NOT HAVING ACHIEVED REMISSION  DIFFUSE LARGE B-CELL LYMPHOMA, EXTRANODAL AND SOLID ORGAN SITES  DIFFUSE LARGE B-CELL LYMPHOMA, UNSPECIFIED SITE  ELEVATED CANCER ANTIGEN 125 [CA 125]  FOLLICULAR LYMPHOMA GRADE I, UNSPECIFIED SITE  FOLLICULAR LYMPHOMA GRADE III, UNSPECIFIED, UNSPECIFIED SITE  FOLLICULAR LYMPHOMA GRADE IIIA, LYMPH NODES OF HEAD, FACE, AND NECK  FOLLICULAR LYMPHOMA GRADE IIIA, UNSPECIFIED SITE  FOLLICULAR LYMPHOMA GRADE IIIB, LYMPH NODES OF MULTIPLE SITES  FOLLICULAR LYMPHOMA, UNSPECIFIED, UNSPECIFIED SITE  HODGKIN LYMPHOMA, UNSPECIFIED, UNSPECIFIED SITE  INTRAHEPATIC BILE DUCT CARCINOMA  LEUKEMIA, UNSPECIFIED NOT HAVING ACHIEVED REMISSION  LIVER CELL CARCINOMA  LYMPHOBLASTIC (DIFFUSE) LYMPHOMA, UNSPECIFIED SITE  MALIGNANT (PRIMARY) NEOPLASM, UNSPECIFIED  MALIGNANT CARCINOID TUMOR OF THE RECTUM  MALIGNANT NEOPLASM ASSOCIATED WITH TRANSPLANTED ORGAN  MALIGNANT NEOPLASM OF ANAL CANAL  MALIGNANT NEOPLASM OF ANUS, UNSPECIFIED  MALIGNANT NEOPLASM OF ASCENDING COLON  MALIGNANT NEOPLASM OF BASE OF TONGUE  MALIGNANT NEOPLASM OF BLADDER, UNSPECIFIED  MALIGNANT NEOPLASM OF CECUM  MALIGNANT NEOPLASM OF CENTRAL PORTION OF RIGHT FEMALE BREAST  MALIGNANT NEOPLASM OF CERVIX UTERI, UNSPECIFIED  MALIGNANT NEOPLASM OF COLON, UNSPECIFIED  MALIGNANT NEOPLASM OF CONNECTIVE AND SOFT TISSUE, UNSPECIFIED  MALIGNANT NEOPLASM OF DESCENDING COLON  MALIGNANT NEOPLASM OF ENDOMETRIUM  MALIGNANT NEOPLASM OF EXOCERVIX  MALIGNANT NEOPLASM OF GALLBLADDER  MALIGNANT NEOPLASM OF GLOTTIS  MALIGNANT NEOPLASM OF HEAD OF PANCREAS  MALIGNANT NEOPLASM OF HEAD, FACE AND NECK  MALIGNANT NEOPLASM OF HEPATIC FLEXURE  MALIGNANT NEOPLASM OF LARYNX, UNSPECIFIED  MALIGNANT NEOPLASM OF LEFT OVARY  MALIGNANT NEOPLASM OF LOWER LOBE, LEFT BRONCHUS OR LUNG  MALIGNANT NEOPLASM OF LOWER LOBE, RIGHT BRONCHUS OR LUNG  MALIGNANT NEOPLASM OF LOWER-INNER QUADRANT OF RIGHT FEMALE BREAST  MALIGNANT NEOPLASM OF MIDDLE LOBE, BRONCHUS OR LUNG  MALIGNANT NEOPLASM OF OROPHARYNX, UNSPECIFIED  MALIGNANT NEOPLASM OF OVERLAPPING SITES OF COLON  MALIGNANT NEOPLASM OF OVERLAPPING SITES OF CORPUS UTERI  MALIGNANT NEOPLASM OF OVERLAPPING SITES OF RECTUM, ANUS AND ANAL CANAL  MALIGNANT NEOPLASM OF PANCREAS, UNSPECIFIED  MALIGNANT NEOPLASM OF PHARYNX, UNSPECIFIED  MALIGNANT NEOPLASM OF PROSTATE  MALIGNANT NEOPLASM OF RECTOSIGMOID JUNCTION  MALIGNANT NEOPLASM OF RECTUM  MALIGNANT NEOPLASM OF RIGHT KIDNEY, EXCEPT RENAL PELVIS  MALIGNANT NEOPLASM OF RIGHT MAIN BRONCHUS  MALIGNANT NEOPLASM OF RIGHT OVARY  MALIGNANT NEOPLASM OF RIGHT UPPER LIMB  MALIGNANT NEOPLASM OF SIGMOID COLON  MALIGNANT NEOPLASM OF SPHENOID SINUS  MALIGNANT NEOPLASM OF SPLENIC FLEXURE  MALIGNANT NEOPLASM OF STOMACH, UNSPECIFIED  MALIGNANT NEOPLASM OF SUPRAGLOTTIS  MALIGNANT NEOPLASM OF THYMUS  MALIGNANT NEOPLASM OF THYROID GLAND  MALIGNANT NEOPLASM OF TRANSVERSE COLON  MALIGNANT NEOPLASM OF TRIGONE OF BLADDER  MALIGNANT NEOPLASM OF UNSPECIFIED KIDNEY, EXCEPT RENAL PELVIS  MALIGNANT NEOPLASM OF UNSPECIFIED OVARY  MALIGNANT NEOPLASM OF UNSPECIFIED PART OF LEFT BRONCHUS OR LUNG  MALIGNANT NEOPLASM OF UNSPECIFIED PART OF RIGHT BRONCHUS OR LUNG  MALIGNANT NEOPLASM OF UNSPECIFIED PART OF UNSPECIFIED BRONCHUS OR LUNG  MALIGNANT NEOPLASM OF UNSPECIFIED SITE OF LEFT FEMALE BREAST  MALIGNANT NEOPLASM OF UNSPECIFIED SITE OF RIGHT FEMALE BREAST  MALIGNANT NEOPLASM OF UNSPECIFIED SITE OF UNSPECIFIED FEMALE BREAST  MALIGNANT NEOPLASM OF UPPER LOBE, LEFT BRONCHUS OR LUNG  MALIGNANT NEOPLASM OF UPPER LOBE, RIGHT BRONCHUS OR LUNG  MALIGNANT NEOPLASM OF UPPER LOBE, UNSPECIFIED BRONCHUS OR LUNG  MALIGNANT NEOPLASM OF URINARY ORGAN, UNSPECIFIED  MALIGNANT NEOPLASM OF UTERUS, PART UNSPECIFIED  MALIGNANT NEOPLASM OF VULVA, UNSPECIFIED  MANTLE CELL LYMPHOMA, INTRA-ABDOMINAL LYMPH NODES  MANTLE CELL LYMPHOMA, LYMPH NODES OF MULTIPLE SITES  MANTLE CELL LYMPHOMA, UNSPECIFIED SITE  MESOTHELIOMA OF OTHER SITES  MESOTHELIOMA OF PLEURA  MESOTHELIOMA, UNSPECIFIED  MULTIPLE MYELOMA NOT HAVING ACHIEVED REMISSION  MYELOID LEUKEMIA, UNSPECIFIED, NOT HAVING ACHIEVED REMISSION  NODULAR SCLEROSIS HODGKIN LYMPHOMA, EXTRANODAL AND SOLID ORGAN SITES  NODULAR SCLEROSIS HODGKIN LYMPHOMA, INTRATHORACIC LYMPH NODES  NODULAR SCLEROSIS HODGKIN LYMPHOMA, UNSPECIFIED SITE  NON-HODGKIN LYMPHOMA, UNSPECIFIED, UNSPECIFIED SITE  OTHER HODGKIN LYMPHOMA, UNSPECIFIED SITE  OTHER MALIGNANT NEUROENDOCRINE TUMORS  OTHER SPECIFIED TYPES OF NON-HODGKIN LYMPHOMA, EXTRANODAL AND SOLID ORGAN SITES  OTHER SPECIFIED TYPES OF NON-HODGKIN LYMPHOMA, LYMPH NODES OF HEAD, FACE, AND NECK  OTHER SPECIFIED TYPES OF NON-HODGKIN LYMPHOMA, UNSPECIFIED SITE  PERSONAL HISTORY OF MALIGNANT NEOPLASM OF BLADDER  PERSONAL HISTORY OF MALIGNANT NEOPLASM OF BRAIN  PERSONAL HISTORY OF MALIGNANT NEOPLASM OF BREAST  PERSONAL HISTORY OF MALIGNANT NEOPLASM OF CERVIX UTERI  PERSONAL HISTORY OF MALIGNANT NEOPLASM OF LARYNX  PERSONAL HISTORY OF MALIGNANT NEOPLASM OF LIVER  PERSONAL HISTORY OF MALIGNANT NEOPLASM OF NASAL CAVITIES, MIDDLE EAR, AND ACCESSORY SINUSES  PERSONAL HISTORY OF MALIGNANT NEOPLASM OF OTHER FEMALE GENITAL ORGANS  PERSONAL HISTORY OF MALIGNANT NEOPLASM OF OTHER ORGANS AND SYSTEMS  PERSONAL HISTORY OF MALIGNANT NEOPLASM OF OTHER PARTS OF UTERUS  PERSONAL HISTORY OF MALIGNANT NEOPLASM OF OTHER SITES OF LIP, ORAL CAVITY, AND PHARYNX  PERSONAL HISTORY OF MALIGNANT NEOPLASM OF OVARY  PERSONAL HISTORY OF MALIGNANT NEOPLASM OF PANCREAS  PERSONAL HISTORY OF MALIGNANT NEOPLASM OF PROSTATE  PERSONAL HISTORY OF MALIGNANT NEOPLASM OF RENAL PELVIS  PERSONAL HISTORY OF MALIGNANT NEOPLASM OF SOFT TISSUE  PERSONAL HISTORY OF MALIGNANT NEOPLASM OF TESTIS  PERSONAL HISTORY OF MALIGNANT NEOPLASM OF THYROID  PERSONAL HISTORY OF MALIGNANT NEOPLASM OF TONGUE  PERSONAL HISTORY OF MALIGNANT NEOPLASM OF UNSPECIFIED SITE OF LIP, ORAL CAVITY, AND PHARYNX  PERSONAL HISTORY OF MALIGNANT NEOPLASM, UNSPECIFIED  PROLYMPHOCYTIC LEUKEMIA OF T-CELL TYPE NOT HAVING ACHIEVED REMISSION  SECONDARY AND UNSPECIFIED MALIGNANT NEOPLASM OF LYMPH NODE, UNSPECIFIED  SECONDARY AND UNSPECIFIED MALIGNANT NEOPLASM OF LYMPH NODES OF HEAD, FACE AND NECK  SECONDARY MALIGNANT NEOPLASM OF BONE  SECONDARY MALIGNANT NEOPLASM OF BRAIN  SECONDARY MALIGNANT NEOPLASM OF GENITAL ORGANS  SECONDARY MALIGNANT NEOPLASM OF LEFT KIDNEY AND RENAL PELVIS  SECONDARY MALIGNANT NEOPLASM OF LEFT LUNG  SECONDARY MALIGNANT NEOPLASM OF LIVER AND INTRAHEPATIC BILE DUCT  SECONDARY MALIGNANT NEOPLASM OF MEDIASTINUM  SECONDARY MALIGNANT NEOPLASM OF OTHER SPECIFIED SITES  SECONDARY MALIGNANT NEOPLASM OF RETROPERITONEUM AND PERITONEUM  SECONDARY MALIGNANT NEOPLASM OF RIGHT ADRENAL GLAND  SECONDARY MALIGNANT NEOPLASM OF RIGHT KIDNEY AND RENAL PELVIS  SECONDARY MALIGNANT NEOPLASM OF RIGHT LUNG  SECONDARY MALIGNANT NEOPLASM OF UNSPECIFIED LUNG  SECONDARY MALIGNANT NEOPLASM OF UNSPECIFIED SITE  SQUAMOUS CELL CARCINOMA OF ANAL SKIN  SQUAMOUS CELL CARCINOMA OF SKIN OF OTHER PARTS OF FACE  SQUAMOUS CELL CARCINOMA OF SKIN OF RIGHT UPPER LIMB, INCLUDING SHOULDER  SQUAMOUS CELL CARCINOMA OF SKIN OF UNSPECIFIED PARTS OF FACE  UNSPECIFIED B-CELL LYMPHOMA, UNSPECIFIED SITE  UNSPECIFIED MALIGNANT NEOPLASM OF SKIN, UNSPECIFIED |
|  | CONDITION | ACUTE LYMPHOBLASTIC LEUKEMIA NOT HAVING ACHIEVED REMISSION  ACUTE MYELOBLASTIC LEUKEMIA, NOT HAVING ACHIEVED REMISSION  ACUTE PROMYELOCYTIC LEUKEMIA, NOT HAVING ACHIEVED REMISSION  AGRANULOCYTOSIS SECONDARY TO CANCER CHEMOTHERAPY  BASAL CELL CARCINOMA OF SKIN OF NOSE  BASAL CELL CARCINOMA OF SKIN OF OTHER PARTS OF FACE  CHRONIC LYMPHOCYTIC LEUKEMIA OF B-CELL TYPE NOT HAVING ACHIEVED REMISSION  CHRONIC MYELOID LEUKEMIA, BCR/ABL-POSITIVE, IN REMISSION  CHRONIC MYELOID LEUKEMIA, BCR/ABL-POSITIVE, NOT HAVING ACHIEVED REMISSION  DIFFUSE LARGE B-CELL LYMPHOMA, EXTRANODAL AND SOLID ORGAN SITES  FOLLICULAR LYMPHOMA, UNSPECIFIED, UNSPECIFIED SITE  HODGKIN LYMPHOMA, UNSPECIFIED, UNSPECIFIED SITE  INTRAHEPATIC BILE DUCT CARCINOMA  LEUKEMIA, UNSPECIFIED NOT HAVING ACHIEVED REMISSION  LIVER CELL CARCINOMA  MALIGNANT (PRIMARY) NEOPLASM, UNSPECIFIED  MALIGNANT NEOPLASM OF ASCENDING COLON  MALIGNANT NEOPLASM OF BLADDER, UNSPECIFIED  MALIGNANT NEOPLASM OF BRAIN, UNSPECIFIED  MALIGNANT NEOPLASM OF CECUM  MALIGNANT NEOPLASM OF CERVIX UTERI, UNSPECIFIED  MALIGNANT NEOPLASM OF COLON, UNSPECIFIED  MALIGNANT NEOPLASM OF ENDOMETRIUM  MALIGNANT NEOPLASM OF LARYNX, UNSPECIFIED  MALIGNANT NEOPLASM OF LIVER, NOT SPECIFIED AS PRIMARY OR SECONDARY  MALIGNANT NEOPLASM OF LOWER LOBE, UNSPECIFIED BRONCHUS OR LUNG  MALIGNANT NEOPLASM OF LOWER-INNER QUADRANT OF RIGHT FEMALE BREAST  MALIGNANT NEOPLASM OF OVERLAPPING SITES OF RECTUM, ANUS AND ANAL CANAL  MALIGNANT NEOPLASM OF PANCREAS, UNSPECIFIED  MALIGNANT NEOPLASM OF PHARYNX, UNSPECIFIED  MALIGNANT NEOPLASM OF PROSTATE  MALIGNANT NEOPLASM OF RECTUM  MALIGNANT NEOPLASM OF RIGHT KIDNEY, EXCEPT RENAL PELVIS  MALIGNANT NEOPLASM OF STOMACH, UNSPECIFIED  MALIGNANT NEOPLASM OF UNSPECIFIED KIDNEY, EXCEPT RENAL PELVIS  MALIGNANT NEOPLASM OF UNSPECIFIED PART OF RIGHT BRONCHUS OR LUNG  MALIGNANT NEOPLASM OF UNSPECIFIED PART OF UNSPECIFIED BRONCHUS OR LUNG  MALIGNANT NEOPLASM OF UNSPECIFIED SITE OF UNSPECIFIED FEMALE BREAST  MALIGNANT NEOPLASM OF UNSPECIFIED TESTIS, UNSPECIFIED WHETHER DESCENDED OR UNDESCENDED  MALIGNANT NEOPLASM OF UPPER LOBE, LEFT BRONCHUS OR LUNG  MALIGNANT NEOPLASM OF UTERUS, PART UNSPECIFIED  MALIGNANT NEOPLASM OF VULVA, UNSPECIFIED  MULTIPLE MYELOMA NOT HAVING ACHIEVED REMISSION  NODULAR SCLEROSIS HODGKIN LYMPHOMA, UNSPECIFIED SITE  NON-HODGKIN LYMPHOMA, UNSPECIFIED, UNSPECIFIED SITE  OTHER SPECIFIED TYPES OF NON-HODGKIN LYMPHOMA, LYMPH NODES OF HEAD, FACE, AND NECK  PERSONAL HISTORY OF MALIGNANT NEOPLASM OF BLADDER  PERSONAL HISTORY OF MALIGNANT NEOPLASM OF BREAST  PERSONAL HISTORY OF MALIGNANT NEOPLASM OF LARYNX  PERSONAL HISTORY OF MALIGNANT NEOPLASM OF LIVER  PERSONAL HISTORY OF MALIGNANT NEOPLASM OF OTHER FEMALE GENITAL ORGANS  PERSONAL HISTORY OF MALIGNANT NEOPLASM OF OTHER ORGANS AND SYSTEMS  PERSONAL HISTORY OF MALIGNANT NEOPLASM OF OTHER PARTS OF UTERUS  PERSONAL HISTORY OF MALIGNANT NEOPLASM OF OTHER SITES OF LIP, ORAL CAVITY, AND PHARYNX  PERSONAL HISTORY OF MALIGNANT NEOPLASM OF PANCREAS  PERSONAL HISTORY OF MALIGNANT NEOPLASM OF PROSTATE  PERSONAL HISTORY OF MALIGNANT NEOPLASM OF THYROID  PERSONAL HISTORY OF MALIGNANT NEOPLASM OF UNSPECIFIED SITE OF LIP, ORAL CAVITY, AND PHARYNX  PROLYMPHOCYTIC LEUKEMIA OF T-CELL TYPE NOT HAVING ACHIEVED REMISSION  SECONDARY MALIGNANT NEOPLASM OF BONE  SECONDARY MALIGNANT NEOPLASM OF BRAIN  SECONDARY MALIGNANT NEOPLASM OF LIVER AND INTRAHEPATIC BILE DUCT  SECONDARY MALIGNANT NEOPLASM OF MEDIASTINUM  SECONDARY MALIGNANT NEOPLASM OF UNSPECIFIED LUNG  SQUAMOUS CELL CARCINOMA OF SKIN OF UNSPECIFIED PARTS OF FACE  UNSPECIFIED MALIGNANT NEOPLASM OF SKIN, UNSPECIFIED |
| Dementia | DIAGNOSIS | ALCOHOL DEPENDENCE WITH ALCOHOL-INDUCED PERSISTING DEMENTIA  ALZHEIMER'S DISEASE WITH EARLY ONSET  ALZHEIMER'S DISEASE WITH LATE ONSET  ALZHEIMER'S DISEASE, UNSPECIFIED  DEMENTIA IN OTHER DISEASES CLASSIFIED ELSEWHERE WITH BEHAVIORAL DISTURBANCE  DEMENTIA IN OTHER DISEASES CLASSIFIED ELSEWHERE WITHOUT BEHAVIORAL DISTURBANCE  DEMENTIA WITH LEWY BODIES  OTHER ALZHEIMER'S DISEASE  OTHER FRONTOTEMPORAL DEMENTIA  UNSPECIFIED DEMENTIA WITH BEHAVIORAL DISTURBANCE  UNSPECIFIED DEMENTIA WITHOUT BEHAVIORAL DISTURBANCE  VASCULAR DEMENTIA WITH BEHAVIORAL DISTURBANCE  VASCULAR DEMENTIA WITHOUT BEHAVIORAL DISTURBANCE |
|  | CONDITION | ALZHEIMER'S DISEASE WITH LATE ONSET  ALZHEIMER'S DISEASE, UNSPECIFIED  DEMENTIA IN OTHER DISEASES CLASSIFIED ELSEWHERE WITH BEHAVIORAL DISTURBANCE  DEMENTIA IN OTHER DISEASES CLASSIFIED ELSEWHERE WITHOUT BEHAVIORAL DISTURBANCE  UNSPECIFIED DEMENTIA WITH BEHAVIORAL DISTURBANCE  UNSPECIFIED DEMENTIA WITHOUT BEHAVIORAL DISTURBANCE  VASCULAR DEMENTIA WITHOUT BEHAVIORAL DISTURBANCE |
| No intubation but respiratory failure, hypoxemia or dependence on supplemental oxygen | DIAGNOSIS | DEPENDENCE ON SUPPLEMENTAL OXYGEN  RESPIRATORY FAILURE  HYPOXEMIA |
|  | CONDITION | DEPENDENCE ON SUPPLEMENTAL OXYGEN  RESPIRATORY FAILURE  HYPOXEMIA |
| Intubation | DIAGNOSIS | DEPENDENCE ON RESPIRATOR [VENTILATOR] STATUS  FAILED OR DIFFICULT INTUBATION, INITIAL ENCOUNTER  VENTILATOR ASSOCIATED PNEUMONIA |
|  | CONDITION | VENTILATOR ASSOCIATED PNEUMONIA |
|  | PROCEDURES | ASSISTANCE WITH RESPIRATORY VENTILATION  RESPIRATORY VENTILATION |
| Pneumonia | CONDITION | INFLUENZA DUE TO OTHER IDENTIFIED INFLUENZA VIRUS WITH OTHER SPECIFIED PNEUMONIA  INFLUENZA DUE TO OTHER IDENTIFIED INFLUENZA VIRUS WITH UNSPECIFIED TYPE OF PNEUMONIA  KLEBSIELLA PNEUMONIAE [K. PNEUMONIAE] AS THE CAUSE OF DISEASES CLASSIFIED ELSEWHERE  OTHER VIRAL PNEUMONIA  PERSONAL HISTORY OF PNEUMONIA (RECURRENT)  PNEUMONIA DUE TO CORONAVIRUS DISEASE 2019  PNEUMONIA DUE TO OTHER GRAM-NEGATIVE BACTERIA  PNEUMONIA DUE TO PSEUDOMONAS  PNEUMONIA DUE TO SARS-ASSOCIATED CORONAVIRUS  PNEUMONIA DUE TO STREPTOCOCCUS PNEUMONIAE  PNEUMONIA, UNSPECIFIED ORGANISM  SEPSIS DUE TO STREPTOCOCCUS PNEUMONIAE  UNSPECIFIED BACTERIAL PNEUMONIA  VENTILATOR ASSOCIATED PNEUMONIA  VIRAL PNEUMONIA, UNSPECIFIED |
|  | DIAGNOSIS | ABSCESS OF LUNG WITH PNEUMONIA  CRYPTOGENIC ORGANIZING PNEUMONIA  HUMAN METAPNEUMOVIRUS PNEUMONIA  INFLUENZA DUE TO OTHER IDENTIFIED INFLUENZA VIRUS WITH OTHER SPECIFIED PNEUMONIA  INFLUENZA DUE TO OTHER IDENTIFIED INFLUENZA VIRUS WITH UNSPECIFIED TYPE OF PNEUMONIA  INFLUENZA DUE TO UNIDENTIFIED INFLUENZA VIRUS WITH SPECIFIED PNEUMONIA  KLEBSIELLA PNEUMONIAE [K. PNEUMONIAE] AS THE CAUSE OF DISEASES CLASSIFIED ELSEWHERE  LOBAR PNEUMONIA, UNSPECIFIED ORGANISM  OTHER PNEUMONIA, UNSPECIFIED ORGANISM  OTHER VIRAL PNEUMONIA  PERSONAL HISTORY OF PNEUMONIA (RECURRENT)  PNEUMONIA DUE TO CORONAVIRUS DISEASE 2019  PNEUMONIA DUE TO ESCHERICHIA COLI  PNEUMONIA DUE TO KLEBSIELLA PNEUMONIAE  PNEUMONIA DUE TO METHICILLIN RESISTANT STAPHYLOCOCCUS AUREUS  PNEUMONIA DUE TO METHICILLIN SUSCEPTIBLE STAPHYLOCOCCUS AUREUS  PNEUMONIA DUE TO OTHER GRAM-NEGATIVE BACTERIA  PNEUMONIA DUE TO OTHER SPECIFIED BACTERIA  PNEUMONIA DUE TO OTHER SPECIFIED INFECTIOUS ORGANISMS  PNEUMONIA DUE TO PSEUDOMONAS  PNEUMONIA DUE TO SARS-ASSOCIATED CORONAVIRUS  PNEUMONIA DUE TO STAPHYLOCOCCUS, UNSPECIFIED  PNEUMONIA DUE TO STREPTOCOCCUS PNEUMONIAE  PNEUMONIA, UNSPECIFIED ORGANISM  SEPSIS DUE TO STREPTOCOCCUS PNEUMONIAE  UNSPECIFIED BACTERIAL PNEUMONIA  VENTILATOR ASSOCIATED PNEUMONIA  VIRAL PNEUMONIA, UNSPECIFIED |
| Sepsis | CONDITION | OTHER SPECIFIED SEPSIS  OTHER STREPTOCOCCAL SEPSIS  SEPSIS DUE TO ANAEROBES  SEPSIS DUE TO ENTEROCOCCUS  SEPSIS DUE TO METHICILLIN RESISTANT STAPHYLOCOCCUS AUREUS  SEPSIS DUE TO OTHER SPECIFIED STAPHYLOCOCCUS  SEPSIS DUE TO SERRATIA  SEPSIS DUE TO STREPTOCOCCUS PNEUMONIAE  SEPSIS DUE TO UNSPECIFIED STAPHYLOCOCCUS  SEPSIS, UNSPECIFIED ORGANISM  SEPTIC ARTERIAL EMBOLISM  SEVERE SEPSIS WITH SEPTIC SHOCK  SEVERE SEPSIS WITHOUT SEPTIC SHOCK |
|  | DIAGNOSIS | OTHER SPECIFIED SEPSIS  SEVERE SEPSIS WITH SEPTIC SHOCK  SEPSIS, UNSPECIFIED ORGANISM  SEPSIS DUE TO ESCHERICHIA COLI [E. COLI]  SEPSIS DUE TO METHICILLIN RESISTANT STAPHYLOCOCCUS AUREUS  SEPSIS DUE TO OTHER SPECIFIED STAPHYLOCOCCUS  SEVERE SEPSIS WITHOUT SEPTIC SHOCK  SEPSIS DUE TO ENTEROCOCCUS  SEPTIC PULMONARY EMBOLISM WITHOUT ACUTE COR PULMONALE  SEPSIS DUE TO METHICILLIN SUSCEPTIBLE STAPHYLOCOCCUS AUREUS  STREPTOCOCCAL SEPSIS, UNSPECIFIED  OTHER GRAM-NEGATIVE SEPSIS  SEPTIC ARTERIAL EMBOLISM  SEPSIS FOLLOWING A PROCEDURE, INITIAL ENCOUNTER  GRAM-NEGATIVE SEPSIS, UNSPECIFIED  SEPSIS DUE TO PSEUDOMONAS  OTHER STREPTOCOCCAL SEPSIS  CANDIDAL SEPSIS  SEPSIS DUE TO ANAEROBES  SEPSIS DUE TO SERRATIA  SEPSIS DUE TO STREPTOCOCCUS PNEUMONIAE |
| Obesity | CONDITION | MORBID (SEVERE) OBESITY DUE TO EXCESS CALORIES  MORBID (SEVERE) OBESITY WITH ALVEOLAR HYPOVENTILATION  OBESITY COMPLICATING CHILDBIRTH  OBESITY COMPLICATING PREGNANCY, THIRD TRIMESTER  OBESITY, UNSPECIFIED  OTHER OBESITY DUE TO EXCESS CALORIES |
|  | DIAGNOSIS | MORBID (SEVERE) OBESITY DUE TO EXCESS CALORIES  MORBID (SEVERE) OBESITY WITH ALVEOLAR HYPOVENTILATION  OBESITY COMPLICATING CHILDBIRTH  OBESITY COMPLICATING PREGNANCY, FIRST TRIMESTER  OBESITY COMPLICATING PREGNANCY, SECOND TRIMESTER  OBESITY COMPLICATING PREGNANCY, THIRD TRIMESTER  OBESITY COMPLICATING PREGNANCY, UNSPECIFIED TRIMESTER  OBESITY, UNSPECIFIED  OTHER OBESITY  OTHER OBESITY DUE TO EXCESS CALORIES |
|  | VITAL | ORIGINAL_BMI ≥30 |
| Steroids | MED_ADMIN | BETAMETHASONE  DEFLAZACORT  DEXAMETHASONE  FLUDROCORTISONE  HYDROCORTISONE  METHYLPREDNISOLONE  PREDNISOLONE  PREDNISONE  TRIAMCINOLONE |
|  | PRESCRIBING | BETAMETHASONE  DEFLAZACORT  DEXAMETHASONE  FLUDROCORTISONE  HYDROCORTISONE  METHYLPREDNISOLONE  PREDNISOLONE  PREDNISONE  TRIAMCINOLONE |
|  | PROCEDURES | BETAMETHASONE  DEFLAZACORT  DEXAMETHASONE  FLUDROCORTISONE  HYDROCORTISONE  METHYLPREDNISOLONE  PREDNISOLONE  PREDNISONE  TRIAMCINOLONE |
| Convalescent plasma | PROCEDURES | TRANSFUSION OF CONVALESCENT PLASMA (NONAUTOLOGOUS) INTO CENTRAL VEIN  TRANSFUSION OF CONVALESCENT PLASMA (NONAUTOLOGOUS) INTO PERIPHERAL VEIN |
| Anticoagulants | MED_ADMIN | APIXABAN  ARGATROBAN  BIVALIRUDIN  DABIGATRAN  DALTEPARIN  DESIRUDIN  EDOXABAN  ENOXAPARIN  FONDAPARINUX  HEPARIN  LEPIRUDIN  RIVAROXABAN  TINZAPARIN  WARFARIN |
|  | PRESCRIBING | APIXABAN  ARGATROBAN  BIVALIRUDIN  DABIGATRAN  DALTEPARIN  DESIRUDIN  EDOXABAN  ENOXAPARIN  FONDAPARINUX  HEPARIN  LEPIRUDIN  RIVAROXABAN  TINZAPARIN  WARFARIN |
|  | PROCEDURES | APIXABAN  ARGATROBAN  BIVALIRUDIN  DABIGATRAN  DALTEPARIN  DESIRUDIN  EDOXABAN  ENOXAPARIN  FONDAPARINUX  HEPARIN  LEPIRUDIN  RIVAROXABAN  TINZAPARIN  WARFARIN |
| Monoclonal antibody | MED_ADMIN | BASILIXIMAB  BEVACIZUMAB  BRENTUXIMAB  CASIRIVIMAB  DARATUMUMAB  DENOSUMAB  OMALIZUMAB  RITUXIMAB  TOCILIZUMAB |
|  | PRESCRIBING | ADALIMUMAB  ALIROCUMAB  BASILIXIMAB  BEVACIZUMAB  BRENTUXIMAB  CASIRIVIMAB  CERTOLIZUMAB  DARATUMUMAB  DENOSUMAB  ERENUMAB  EVOLOCUMAB  IMDEVIMAB  MEPOLIZUMAB  OMALIZUMAB  PEMBROLIZUMAB  RITUXIMAB  TOCILIZUMAB |
|  | PROCEDURES | ATEZOLIZUMAB  BAMLANIVIMAB  BELIMUMAB  BEVACIZUMAB  BLINATUMOMAB  CASIRIVIMAB  IMDEVIMAB  DARATUMUMAB  DENOSUMAB  DURVALUMAB  IMDEVIMAB  INFLIXIMAB  INOTUZUMAB  IPILIMUMAB  NIVOLUMAB  OMALIZUMAB  PEMBROLIZUMAB  RITUXIMAB  SACITUZUMAB  TOCILIZUMAB |
