# Supplementary material for: Real-world evidence of survival benefit of remdesivir: study of 419 propensity score-matched patients hospitalized over the alpha and delta waves of COVID-19 in New Orleans, LA
Source: Front Med (Lausanne). 2024 May 16;11:1390164. doi: 10.3389/fmed.2024.1390164 (PMC11137210; doi:10.3389/fmed.2024.1390164)
Supplement: Supplementary file 4 [file Table_4.DOCX]

**Suppl. Table S4. List of the 22 covariates used for PS calculation.**

|  |  |  |
| --- | --- | --- |
| 1 | Sex | F / M |
| 2 | Age at admission | <65 years; ≥65 years |
| 3 | Race | Black or African American; White; other/unknown/missing/refused to answer |
| 4 | Ethnicity | Hispanic or Latino; Not Hispanic or Latino; other/unknown/missing/refused to answer |
| 5 | Primary payer for COVID-19 admission | Medicare; Medicaid/government/charity/self-pay; commercial; other/missing |
| 6 | Location of residence | New Orleans, Baton Rouge, Hammond, Jackson or Lafayette; other; missing |
| 7 | Charlson Comorbidity Index based on 17 co-morbidities (score range 0 to 29) [link: https://orthotoolkit.com/charlson-comorbidity-index/] | <5; ≥5 |
| 8 | Congestive heart failure, myocardial infarction,  peripheral vascular disease or cerebrovascular disease * | Yes/no |
| 9 | Chronic pulmonary disease * | Yes/no |
| 10 | Diabetes * | Yes/no |
| 11 | Renal disease * | Yes/no |
| 12 | Liver disease or peptic ulcer disease * | Yes/no |
| 13 | Human immunodeficiency virus (HIV)/ acquired immunodeficiency syndrome (AIDS)* | Yes/no |
| 14 | Lymphoma, leukemia or solid tumor * | Yes/no |
| 15 | Dementia | Yes/no |
| 16 | Oxygenation level on or within 5 days prior to day of RDV initiation or corresponding day in patients who did not receive RDV | No intubation / no respiratory failure, hypoxemia or dependence on supplemental oxygen; No intubation/ but respiratory failure hypoxemia or dependence on supplemental oxygen; Intubation |
| 17 | Pneumonia on or within 5 days prior to day of RDV initiation or corresponding day in patients who did not receive RDV | Yes/no |
| 18 | Sepsis on or within 5 days prior to day of RDV initiation or corresponding day in patients who did not receive RDV | Yes/no |
| 19 | Obesity on or within 5 days prior to day of RDV initiation or corresponding day in patients who did not receive RDV | Yes/no |
| 20 | Steroids on or within 5 days prior to day of RDV initiation or corresponding day in patients who did not receive RDV | Yes/no |
| 21 | Anticoagulants on or within 5 days prior to day of RDV initiation or corresponding day in patients who did not receive RDV | Yes/no |
| 22 | Monoclonal antibody on or within 5 days prior to day of RDV initiation or corresponding day in patients who did not receive RDV | Yes/no |

*Selected individual components of the Charlson’s score
